# Structural modeling and docking analysis of canonical and novel resistance-associated missense mutations in Sudanese *Escherichia coli*

**DOI:** 10.1038/s41598-026-39491-7

**Published:** 2026-02-13

**Authors:** Edison Eukun Sage, Sabah A. E. Ibrahim, Mohd Firdaus-Raih, Samah Omer A. Samhoon, Ahmed Abdelghyoum M. Mohamed, Tarig M. E. Ahmed, Moaaz M. Saadaldin, Omnia H. Suliman, Osama Mohamed, Sofia B. Mohamed, Qurashi M. Ali

**Affiliations:** 1 Department of Applied Physics, Faculty of Science and Technology, Universiti Kebangsaan Malaysia, Bangi, 43600 UKM Selangor Malaysia; 2 Bioinformatics and Biostatistics department, National University Biomedical Research Institute, National University-Sudan, Khartoum, Sudan; 3 Faculty of Medicine Biochemistry and Molecular Biology Department, Al Neelain University, Khartoum, Sudan; 4 Al-Neelain Institute for Medical Research, Khartoum, Sudan; 5 Department of Medicine and Surgery, Dubai Medical University, Dubai, UAE; 6 Department of Molecular Biology, National University Biomedical Research Institute, National University-Sudan, Khartoum, Sudan; 7 National University-Sudan, Khartoum, Sudan

**Keywords:** Escherichia coli, Antimicrobial resistance, Missense mutations, Novel variants, Sudanese clinical isolates, Molecular docking, Computational biology and bioinformatics, Drug discovery, Microbiology

## Abstract

Multidrug-resistant *Escherichia coli* represents a growing public health challenge in low-resource settings, where therapeutic options and routine genomic surveillance remain limited. Here, we present a structural bioinformatics analysis of resistance-associated missense mutations identified in Sudanese *E.coli* clinical isolates. Using whole-genome sequencing data from 55 isolates, we performed an integrated in silico analysis of key antibiotic target proteins to characterize missense variation and assess its potential functional consequences. Consensus pathogenicity prediction identified 19 substitutions as likely deleterious, including multiple novel, previously unreported variants clustered in ribosomal protein L22 (rplV), alongside established resistance-associated substitutions in RNA polymerase and topoisomerase IV. These results expand the mutational landscape of resistance-associated targets in Sudanese strains and highlight region-specific patterns of genetic variation. Structure-based modeling and molecular docking revealed mutation-specific effects on predicted antibiotic interactions across multiple targets, including reduced binding for selected topoisomerase inhibitors and heterogeneous effects on macrolide interactions within L22. In contrast, a gyrase variant retained predicted fluoroquinolone binding despite reduced structural stability, suggesting resistance-associated effects beyond direct drug–target interactions. Collectively, these findings underscore the contribution of non-canonical missense mutations to antimicrobial resistance in Sudanese *E.coli* and demonstrate the utility of computational prioritization frameworks to support resistance surveillance and guide targeted experimental validation.

**Supplementary Information:**

The online version contains supplementary material available at 10.1038/s41598-026-39491-7.

## Introduction

Globally, antimicrobial resistance in pathogenic *Escherichia coli* (*E.coli*) has reached alarming levels, substantially compromising the treatment of common infections. Resistance to key antibiotic classes—including fluoroquinolones, macrolides, and rifampicin—has become increasingly prevalent, with approximately 20% of *E.coli* isolates from urinary tract infections exhibiting reduced susceptibility to fluoroquinolones and other first-line agents^1^. Resistance to third-generation cephalosporins, frequently associated with extended-spectrum β-lactamase production, has been reported in approximately 42% of *E.coli* isolates worldwide, and multidrug resistance is now common, with nearly half of isolates in some studies displaying resistance to multiple antibiotic classes^2^. In Sudan, recent clinical investigations indicate that *E.coli* remains the predominant pathogen in urinary tract and other infections, with resistance rates to commonly prescribed fluoroquinolones such as ciprofloxacin and ofloxacin ranging from 55% to 58%^3^. Alarmingly, more than 90% of isolates exhibit multidrug resistance, reflecting widespread antibiotic misuse and limited antimicrobial stewardship^4^. Resistance to first-line agents—including fluoroquinolones, β-lactams, tetracyclines, and trimethoprim–sulfamethoxazole is widespread^3^. Although data on macrolide and rifampicin resistance in Sudanese *E.coli* remain limited, reported co-resistance patterns suggest potential clinical relevance and underscore the need for strengthened molecular surveillance within the Sudanese healthcare context^5^. At the molecular level, antimicrobial resistance in *E.coli* arises through a combination of horizontally acquired resistance determinants and chromosomal mutations affecting antibiotic target proteins. Fluoroquinolone resistance is most commonly mediated by substitutions within the quinolone resistance–determining regions (QRDRs) of gyrA, gyrB, parC, and parE, with well-established examples including gyrA S83L and D87N and parC S80I and E84K^6–10^. Resistance to other antibiotic classes is similarly linked to target-site alterations: aminoglycoside resistance frequently involves mutations in rpsL (e.g., K43R and K88R); macrolide resistance is associated with substitutions or deletions in ribosomal proteins L4 (*rplD*) and ribosomal protein L22 (rplV); and rifampicin resistance is driven primarily by substitutions in rpoB (e.g., S531L, H526Y, and D516V), often accompanied by compensatory mutations in rpoC that mitigate fitness costs^11,12^. Beyond genotypic surveillance, structural and computational approaches have increasingly been used to elucidate how resistance-associated mutations alter protein stability, conformational dynamics, and antibiotic binding in *E.coli* and other bacterial pathogens. Structure-based studies have clarified the molecular basis of fluoroquinolone resistance by mapping QRDR substitutions in DNA gyrase and topoisomerase IV and modeling their effects on drug–DNA–enzyme complexes^13^. Similarly, structural investigations of ribosomal proteins L4 and L22 have demonstrated how mutations reshape the macrolide-binding tunnel, resulting in steric hindrance or altered ligand accommodation^10^. For RNA polymerase, crystallographic and modeling studies have shown that rpoB mutations disrupt rifampicin binding while preserving transcriptional activity, often through subtle conformational changes rather than large-scale structural rearrangements^14^. Despite these advances, most prior structural analyses have focused on canonical resistance mutations, laboratory strains, or globally prevalent variants. Consequently, the structural and functional consequences of geographically specific or non-canonical missense substitutionsparticularly those emerging in underrepresented regions—remain insufficiently characterized. This gap is especially pronounced for *E.coli* isolates from low- and middle-income countries, including Sudan, where routine genomic surveillance is limited and resistance evolution may follow region-specific trajectories. Although whole-genome sequencing and computational prediction frameworks have improved resistance detection and phenotype inference^15,16^, they often provide limited insight into how individual missense mutations influence protein structure, stability, and antibiotic–target interactions, especially for variants outside classical QRDR hotspots. Structural bioinformatics offers a complementary framework to address this limitation by linking sequence variation to three-dimensional protein architecture and drug-binding mechanisms, enabling functional interpretation of both canonical and non-canonical resistance-associated variants^17^. Structure-based analyses of DNA gyrase, topoisomerase IV, ribosomal proteins, and RNA polymerase have demonstrated that resistance can arise through localized perturbations of ligand binding or protein dynamics without necessitating extensive structural rearrangements^18^. However, such approaches have rarely been applied systematically to geographically specific variant sets from underrepresented regions, limiting their integration into antimicrobial resistance surveillance efforts^19^. Previous studies of *E.coli* antimicrobial resistance in Sudan have largely emphasized phenotypic susceptibility patterns, the prevalence of multidrug resistance, and the detection of well-established resistance determinants, particularly canonical QRDR mutations and plasmid-mediated resistance genes^20,21^. While these investigations provide essential epidemiological context, they offer limited insight into how missense variation within antibiotic target proteins affects protein stability, molecular dynamics, or drug–target interactions—especially for non-canonical or previously unreported substitutions. In this study, we address this gap by conducting a comprehensive structural bioinformatics analysis of resistance-associated missense mutations in clinically characterized Sudanese *E.coli* isolates. Using 55 genomes derived from three Sudan-focused BioProjects encompassing hospital-derived, urinary, clinical, and environmental isolates^22–24^, we integrate pathogenicity prediction, evolutionary conservation analysis, structural modeling, and molecular docking to evaluate both canonical and novel variants. Rather than proposing new resistance mechanisms, our objective is to contextualize Sudanese variants within the broader structural literature, expand the known mutational landscape beyond classical QRDR substitutions, and generate mechanistic, testable hypotheses regarding how region-specific missense mutations may influence protein structure, stability, and antibiotic interactions. This framework supports mutation prioritization for experimental validation and contributes to more locally informed antimicrobial resistance surveillance.

## Results

### Distribution of missense mutations across resistance-associated proteins

**Fig. 1:**
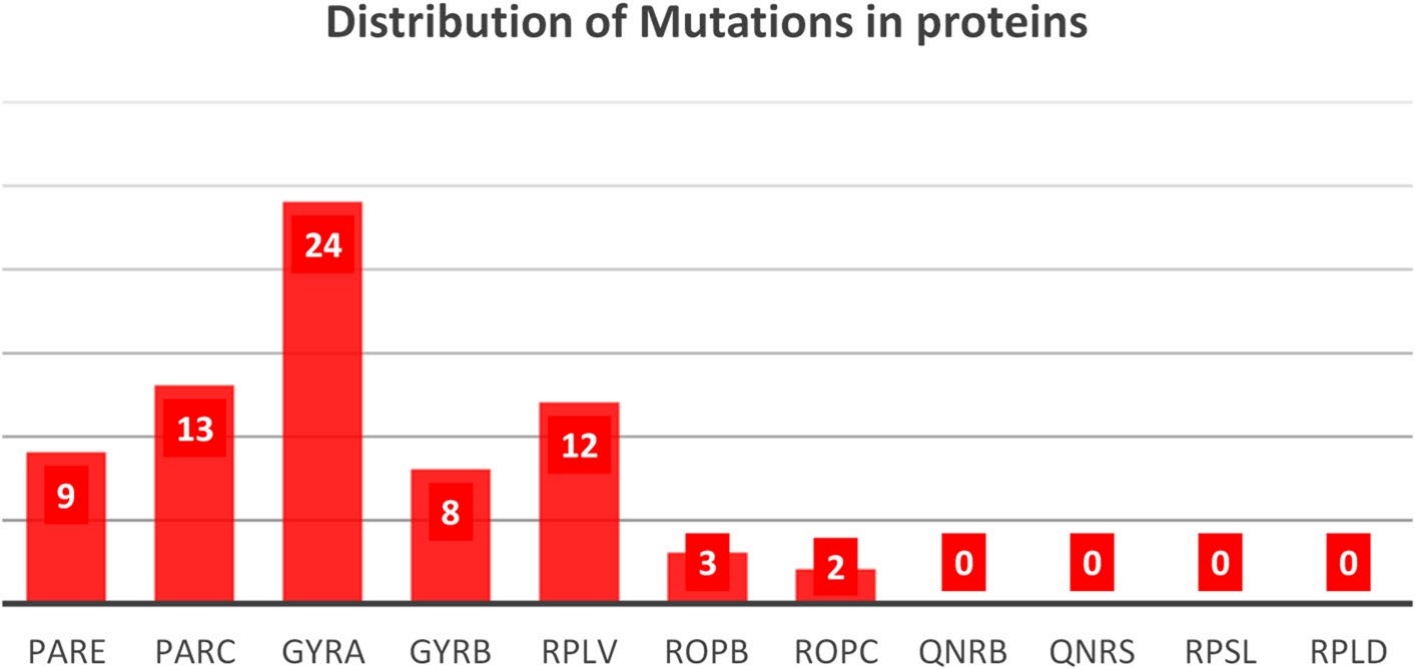
Distribution of missense mutations across resistance-associated proteins in Sudanese *E.coli* isolates. Bar chart showing the number of missense mutations identified in key antibiotic target proteins across 55 Sudanese *E. coli* genomes (total = 71). Mutations were most frequent in GyrA, ParC, and ribosomal protein L22 (rplV), identifying these proteins as major mutation hotspots, while fewer substitutions were observed in RNA polymerase subunits and none in plasmid-mediated quinolone resistance genes (qnrB, qnrS).

A total of 71 mutations were identified across key antibiotic resistance–associated proteins in 55 Sudanese *E.coli* isolates. As shown in Fig. 1, the DNA gyrase subunit A (GyrA; gene gyrA) harbored the highest number of mutations, with 24 substitutions (33.8%), underscoring its central role in fluoroquinolone resistance. The topoisomerase IV subunit ParC (parC) contained 13 mutations (18.3%), while ribosomal protein L22 (rplV) exhibited 12 mutations (16.9%), indicating substantial mutational diversity in both fluoroquinolone- and macrolide-associated targets. Moderate numbers of mutations were observed in ParE (parE) (9 mutations; 12.7%) and the gyrase subunit GyrB (gyrB) (8 mutations; 11.3%). In contrast, relatively few substitutions were detected in RNA polymerase subunits, with RpoB (rpoB) and RpoC (rpoC) showing 3 (4.2%) and 2 (2.8%) mutations, respectively. No mutations were identified in plasmid-mediated quinolone resistance genes (qnrB and qnrS) or in the ribosomal protein genes rpsL and rplD within this dataset. Overall, these findings identify GyrA, GyrB, ParC, and ribosomal protein L22 as the principal mutation hotspots among resistance-associated proteins in Sudanese *E.coli* isolates.

### Functional impact and pathogenicity of missense variants

Among the 71 missense mutations identified across resistance-associated proteins in the analyzed *E.coli* isolates, 19 variants (26.8%) were classified as deleterious and were distributed across five proteins (RpoB, ParE, ParC, GyrA, and ribosomal protein L22). No deleterious mutations were detected in GyrB or RpoC (Supplementary Tables S1–S7). Of the 19 deleterious variants, 13 (68.4%) were classified as novel missense substitutions. The largest number of deleterious novel variants was observed in ribosomal protein L22 (rplV), with all such mutations detected in a single isolate (UMX99540.1). Deleterious substitutions were also identified in established resistance-associated targets, including rpoB (D516N and H526N), parE (D250G and S458A), and parC (E84G and S80I) (Table 1).

**Table 1:** Deleterious canonical and novel missense mutations in resistance-associated proteins of Sudanese *E.coli*.

Protein	Mutation	Isolates with mutation (*n*)	PredictSNP (score)	Category
RpoB	D516N	3	Deleterious (61%)	Canonical (rifampicin resistance)
	H526N	1	Deleterious (55%)	Canonical (rifampicin resistance)
ParE	D250G	1	Deleterious (55%)	Canonical (fluoroquinolone resistance)
	S458A	9	Deleterious (76%)	Canonical (fluoroquinolone resistance)
ParC	L440R	1	Deleterious (87%)	Novel
	S80I	20	Deleterious (64%)	Canonical (fluoroquinolone resistance)
	P577L	1	Deleterious (55%)	Novel
	E84G	1	Deleterious (55%)	Canonical (fluoroquinolone resistance)
GyrA	G214V	1	Deleterious (87%)	Novel
Ribosomal protein L22 (*rplV*)	V71S	1	Deleterious (87%)	Novel
	I74N	1	Deleterious (87%)	Novel
	V76R	1	Deleterious (87%)	Novel
	D77R	1	Deleterious (87%)	Novel
	G79R	1	Deleterious (87%)	Novel
	K83A	1	Deleterious (87%)	Novel
	K83E	1	Deleterious (87%)	Novel
	M82H	1	Deleterious (72%)	Novel
	S81E	1	Deleterious (61%)	Novel
	E78R	1	Deleterious (55%)	Novel

PredictSNP scores represent consensus pathogenicity confidence. Variants were classified as deleterious based on majority agreement across integrated prediction tools. “Canonical” refers to mutations previously associated with antibiotic resistance, while “Novel” indicates variants not previously reported in resistance databases.

### Stability predictions of deleterious missense mutations

The structural stability of deleterious missense mutations was evaluated using five independent computational tools (mCSM, I-Mutant2.0, SDM, DUET, and MuPro). Overall, the majority of deleterious variants were predicted to have a destabilizing effect on protein structure, although the magnitude and direction of ΔΔG values varied across tools (Table 2). Among all analyzed variants, RpLV-V71S consistently exhibited the strongest destabilizing effect, with markedly negative ΔΔG values predicted by mCSM (–2.995 kcal/mol), SDM (–4.39 kcal/mol), DUET (–3.363 kcal/mol), and MuPro (–1.50 kcal/mol), supported by a high Reliability Index (RI = 9) from I-Mutant2.0. Other RpLV substitutions, including I74N, V76R, G79R, and D77R, were also predominantly predicted to destabilize protein structure, indicating a substantial impact of mutations clustered within the ribosomal protein L22. Strong destabilizing effects were additionally observed for ParC-L440R and ParC-E84G, both of which showed consistently negative ΔΔG values across multiple tools. In contrast, several mutations—including RpLV-K83A, K83E, E78R, and S81E—displayed mixed predictions, with some tools suggesting mild stabilization while others, particularly MuPro, predicted an overall loss of stability. These variants were therefore classified as destabilizing based on the consensus rule applied in this study.

Mutations in canonical resistance-associated targets outside RpLV also showed destabilizing trends. RpoB-D516N and RpoB-H526N were predicted to reduce structural stability by most tools, while ParE-D250G and ParE-S458A demonstrated moderate destabilization despite occasional stabilizing predictions from SDM or DUET. The I-Mutant2.0 Reliability Index values ranged from 2 to 9, with higher scores indicating greater confidence in the predicted stability changes (Table 2).

**Table 2:** Predicted effects of deleterious missense mutations on protein stability assessed using multiple computational tools.

Protein	Mutation	mCSM (ΔΔG)	I-Mutant2.0(RI)	SDM(ΔΔG)	DUET (ΔΔG)	MuPro (delta G)
RpoB	D516N	Destabilizing − 0.452	Decrease 8	Destabilizing − 0.03	Destabilizing − 0.222	Decrease (− 0.92220087)
	H526N	Destabilizing − 1.824	Increase 2	Destabilizing − 1.73	Destabilizing − 1.973	Decrease (− 0.93348946)
ParE	D250G	Destabilizing (− 0.996)	Decrease (8)	Stabilizing (1.46)	Destabilizing (− 0.453)	Decrease (− 1.4316958)
	S458A	Destabilizing (− 0.542)	Decrease (7)	Stabilizing (0.047)	Stabilizing (0.047)	Decrease (− 0.90059732)
ParC	L440R	Destabilizing (− 1.792)	Increase (4)	Destabilizing (− 1.76 )	Destabilizing (− 1.737)	Decrease (− 1.665466)
	S80I	Destabilizing − 0.255	Decrease (7.0)	Stabilizing (1.26)	Stabilizing (0.28)	Decrease (− 0.080932255)
	P577L	Destabilizing (− 0.395)	Decrease (7.0)	Stabilizing (0.21)	Destabilizing (− 0.029)	Decrease (− 0.29176239)
	E84G	Destabilizing − 1.222	Decrease (7.0)	Destabilizing − 1.62	Destabilizing − 1.503	Decrease (− 1.2370893)
GyrA	G214V	Destabilizing − 0.09	Decrease (7.0)	Destabilizing − 1.22	Destabilizing − 1.203	Decrease − 0.63080538
Ribosomal protein L22 (rplV)	V71S	Destabilizing − 2.995	Decrease (9)	Destabilizing − 4.39	Destabilizing − 3.363	Decrease − 1.5044668
	I74N	Destabilizing − 1.674	Decrease (8)	Destabilizing − 2.67	Destabilizing − 1.838	Decrease − 1.8920383
	V76R	Destabilizing − 0.91	Decrease 4	Destabilizing − 3.8	Destabilizing − 1.128	Decrease − 2.2445893
	D77R	Destabilizing − 0.412	Decrease 5	Stabilizing 0.48	Destabilizing − 0.143	Decrease − 0.57090402
	G79R	Destabilizing − 0.769	Decrease (6)	Destabilizing − 2.27	Destabilizing − 0.805	Decrease − 0.51340754
	K83A	Stabilizing 0.004	Decrease (7)	Destabilizing − 0.43	Stabilizing 0.068	Decrease − 0.69349162
	K83E	Stabilizing 0.337	Increase 5	Stabilizing 0.43	Stabilizing 0.707	Decrease − 0.63173928
	M82H	Destabilizing − 0.384	Decrease 8	Stabilizing 0.12	Destabilizing − 0.09	Decrease − 1.6917171
	S81E	Destabilizing − 0.067	Decrease 6	Stabilizing 1.01	Stabilizing 0.501	Decrease − 0.19598935
	E78R	Stabilizing 0.111	Decrease 9	Destabilizing − 0.12	Stabilizing 0.268	Decrease − 0.64778912

### Localization of missense mutations within functional domains

Mapping of deleterious and prioritized missense mutations using InterPro revealed that variants were predominantly localized within functionally and structurally critical domains of antibiotic target proteins (Table 3). In rpoB, substitutions D516N and H526N were located within the RNA polymerase β-subunit domain involved in transcriptional elongation and rifampicin binding, consistent with their established role in rifampicin resistance. In the topoisomerase IV subunits, mutations in parE and parC were mapped to conserved domains essential for DNA cleavage and re-ligation. Specifically, parE substitutions D250G and S458A were located within the type IIA topoisomerase and TOPRIM domains, respectively, which are critical for catalytic activity. Similarly, parC mutations—including S80I, E84G, and L440R—were positioned within the DNA topoisomerase type IIA domain A, a well-established hotspot for fluoroquinolone resistance. The gyrA mutation G214V was confined to the DNA gyrase subunit A domain, further supporting its potential role in modulating quinolone–target interactions. Notably, a high density of mutations was observed in RpLV, with multiple substitutions clustered within the ribosomal protein L22 domain. These variants localized to regions lining the nascent peptide exit tunnel and overlapping predicted protein–rRNA interaction sites, which are known to influence macrolide binding and ribosomal function.

Overall, the concentration of missense mutations within conserved functional domains associated with enzymatic activity, structural integrity, or antibiotic interaction supports their potential contribution to antimicrobial resistance. These domain-level annotations provide a structural framework for interpreting downstream stability, dynamics, and molecular docking analyses, and emphasize the importance of experimental validation of non-canonical variants.

**Table 3:** Localization of prioritized deleterious missense mutations within conserved functional domains of resistance-associated *E.coli* proteins.

Protein	Mutations	InterPro Result	Domain
RpoB	D516N H526N	InterPro IPR007645	D/ RNA polymerase Rpb2, domain 3 (513–580)
ParE	D250G	InterPro IPR013506	D/ DNA topoisomerase, type IIA, subunit B, domain 2 (218–384)
	S458A	InterPro IPR006171	D/ TOPRIM domain (412–525)
ParC	L440R	InterPro IPR002205	D/ DNA topoisomerase, type IIA, domain A (8–467)/ Coil
	S80I	InterPro IPR002205 CDD – cd00187	D/ DNA topoisomerase, type IIA, domain A (8–467)/ CAP-like domain
	P577L	InterPro IPR035516	H/ topoisomerase IV, subunit A, C-terminal
	E84G	InterPro IPR002205 CDD – cd00187	D/ DNA topoisomerase, type IIA, domain A (8–467) CAP-like domain
GyrA	G214V	Model: 3.90.199.10-FF-000001	F/ DNA gyrase subunit A 32–281
Ribosomal protein L22 (rplV)	V71S	CDD – cd00336	D/ Ribosomal_L22 (5–107). Putative translocon binding site
	I74N	CDD – cd00336	D/ Ribosomal_L22 (5–107). Putative translocon binding site
	V76R	CDD – cd00336	D/ Ribosomal_L22 (5–107). Protein-rRNA interface
	D77R	CDD – cd00336	D/ Ribosomal_L22 (5–107). Protein-rRNA interface
	G79R	CDD – cd00336	D/ Ribosomal_L22 (5–107). Protein-rRNA interface
	K83A/ K83E	PROSITE Patterns (PS00464) InterPro (IPR018260)	D/ Ribosomal_L22 (5–107). Ribosomal protein L22 signature. (83–107) Large ribosomal subunit protein uL22, conserved site (83–107)
	M82H	CDD – cd00336	D/ Ribosomal_L22 (5–107). Putative translocon binding site
	S81E	CDD cd00336	D/ Ribosomal_L22 (5–107). Putative translocon binding site
	E78R	CDD cd00336	D/ Ribosomal_L22 (5–107). Putative translocon binding site

D (domain), H (Homology), F (family), CDD (Conserved domain database),

### Functional and evolutionary impact of missense mutations

Computational analysis of missense mutations in key *E.coli* proteins revealed a substantial potential to disrupt protein function, stability, and drug interactions. MutPred2 scores for the identified variants ranged from 0.646 to 0.927, with most exceeding the deleterious threshold of 0.5, indicating a high likelihood of functional impairment. Among these, GyrA-G214V exhibited the highest pathogenicity score (0.927), followed by ParC-S80I (0.883) and RpLV variants such as V71S (0.895) and K83A/K83E (~ 0.89), highlighting these as particularly impactful mutations.

ConSurf conservation analysis showed that most mutated residues were highly conserved, with scores ranging from 6 to 9, underscoring their evolutionary and functional importance. Mutations such as RpoB-D516N and H526N, as well as ParE-D250G and S458A, were located in conserved, flexible, or solvent-exposed regions, suggesting that they may influence ligand binding or enzymatic activity. Furthermore, several substitutions overlapped with functional motifs identified by PROSITE and ELM, including ELME000063, PS00005, and ELME000336. Notably, RpoB-H526N and RpLV-K83E/K83A were located within or near conserved motifs critical for structural integrity and molecular interactions, indicating potential disruption of key regulatory or catalytic elements.

MutFunc predictions reinforced these observations, indicating that several variants—such as ParC-L440R and RpLV-G79R—are situated at protein–protein or protein–RNA interfaces, which are essential for ribosome or topoisomerase function. Disruption of such interfaces could compromise bacterial survival and directly affect antibiotic susceptibility (Table 4).

**Table 4:** Conservation and predicted functional impact of prioritized missense mutations in resistance-associated *E. coli* proteins.

Protein	Mutation	Mutpred2			Consurf result	MutFunc
		Score	Remarks	Affected prosite and ELM motives		
RpoB	D516N	0.656	Predicted conservation score	none	Highly conserved (9)/ e/f	Impactful mutations Conservation
	H526N	0.821	Predicted conservation scores	ELME000063, ELME000334, PS00005	Highly conserved (9)/ e/f	Impactful mutations Conservation
ParE	D250G	0.800	Predicted conservation scores	ELME000052	conserved (8)/ e/f	Impactful mutations Conservation
	S458A	0.689	Predicted conservation scores	ELME000117, ELME000202	Highly conserved (9)/ e/f	Impactful mutations Conservation
ParC	L440R	0.851	Predicted conservation scores	ELM00064, PS00005, PS00006	Conserved (7)/ b	Impactful mutations Conservation
	S80I	0.883	Predicted conservation scores	ELME000007, ELM000085	Average conservation (5)/ s	Impactful mutations Conservation
	P577L	0.455	Predicted conservation scores	-	Variable (1)/e	Impactful mutations Conservation
	E84G	0.861	Predicted conservation scores	ELME000120, ELME000317	Conserved (7)/ e	-
GyrA	G214V	0.927	Predicted conservation scores	ELME000155	Conserved (8)/ b	Impactful mutations Stability Conservation
Ribosomal protein L22 (rplV)	V71S	0.895	Predicted conservation scores	ELME000336, PS00005	Conserved (8)/ b	Impactful mutations. Stability.Conservation
	I74N	0.890	Predicted conservation scores	ELME000336	Conserved (6)/ b	Impactful mutations. Stability. Conservation
	V76R	0.863	Predicted conservation scores	None	Conserved (8)/ b	Impactful mutations. Stability. Conservation
	D77R	0.812	Predicted conservation scores	None	Conserved (7)/ e	Impactful mutations .Conservation
	G79R	0.891	Predicted conservation scores	ELME000062, ELME000103	Conserved (6)/ e	Impactful mutations. Interface residues. Conservation
	K83E	0.885	Predicted conservation scores	ELME000100, ELME000108, PS00005, PS00464	Conserved (8)/ e, f	Impactful mutations. Interface residues. Conservation
	K83A	0.896	Predicted conservation scores	ELME000100, ELME000108, PS00005, PS00464	Conserved (8)/ e, f	Impactful mutations. Interface residues. Conservation
	M82H	0.791	Predicted conservation scores	PS00005	Average (5)/ b	Impactful mutations. Interface residues. Conservation
	S81E	0.646	Predicted conservation scores	PS00005	Conserved (6)/ e	Impactful mutations. Interface residues. Conservation
	E78R	0.693	Predicted conservation scores	None	Variable (2)/e	Impactful mutations. Interface residues. Conservation

(e) An exposed residue according to the neural network algorithm. (b) A buried residue according to the neural network algorithm. (f) A predicted functional residue (highly conserved and exposed). (s) A predicted structural residue (highly conserved and buried).


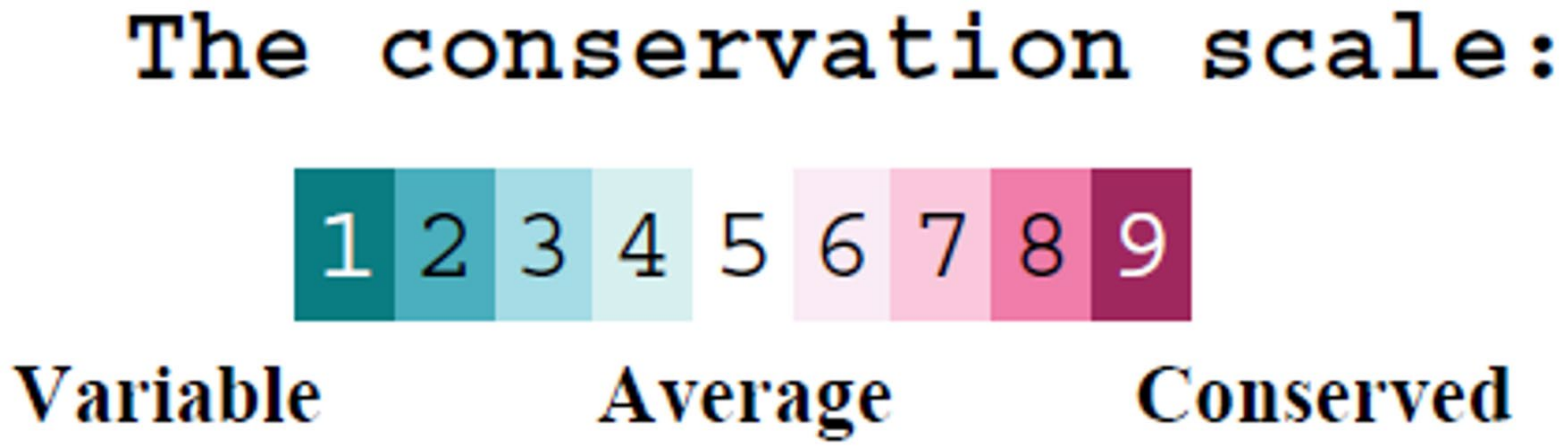


### Impact of missense mutations on protein molecular mechanisms predicted by MutPred2

To evaluate the functional consequences of missense mutations in key *E.coli* proteins, we applied MutPred2 to resistance- and virulence-associated targets including RpoB, ParE, ParC, GyrA, GyrB, RpLV, and RpoC. Several variants exhibited high probability scores (≥ 0.25), indicating a substantial likelihood of disrupting protein function through diverse molecular mechanisms.

For RpoB, the canonical mutation D516N was predicted to alter disordered interface regions, while H526N gained a novel allosteric site at R529 and methylation at K527, suggesting altered RNA polymerase activity and transcriptional regulation. ParE mutations such as D250G and S458A were predicted to destabilize helices, alter ordered interfaces, and disrupt allosteric and catalytic sites, providing a mechanistic basis for fluoroquinolone resistance. Similarly, ParC variants L440R, S80I, and E84G were associated with ordered/disordered interface disruption, gain or loss of catalytic/allosteric sites, and altered solvent accessibility. In contrast, P577L did not exhibit significant predicted effects.

The GyrA G214V substitution was associated with loss of a catalytic site (T219) and altered solvent accessibility, consistent with quinolone resistance. Novel RpLV variants (V71S, I74N, V76R, D77R, E78R, G79R, K83A, K83E, M82H, and S81E) exhibited recurrent patterns of disrupted structural interfaces, altered solvent accessibility, metal-binding changes, and gain/loss of acetylation and catalytic sites, all within the L22 macrolide-binding tunnel, suggesting a role in ribosome-mediated resistance.

Overall, the most frequently impacted molecular features across all mutations included altered ordered/disordered interfaces, loss of solvent accessibility, gain/loss of catalytic and allosteric sites, and secondary structure destabilization (Table S8).

### Predicted effects of missense mutations on protein–protein interaction affinity (mCSM-PPI analysis)

The impact of missense mutations on protein–protein interaction (PPI) affinity was evaluated using the mCSM-PPI tool, which predicts changes in binding free energy (ΔΔG), with negative values indicating reduced affinity (destabilization) and positive values indicating enhanced affinity (stabilization). Among RNA polymerase variants, RpoB D516N exhibited a marked decrease in binding affinity (ΔΔG = − 0.54 kcal/mol), consistent with significant destabilization of RNA polymerase complex interactions, whereas H526N showed a moderate reduction (ΔΔG = − 0.27 kcal/mol), suggesting a milder effect on complex assembly.

All analyzed ParE and ParC mutations yielded ΔΔG values of 0.00 kcal/mol, indicating no predicted impact on protein–protein interaction affinity and suggesting preservation of topoisomerase IV subunit assembly. Importantly, PPI neutrality in this context indicates preservation of inter-subunit assembly and does not preclude functional disruption mediated through alternative mechanisms, such as local structural destabilization, altered conformational dynamics, or impaired drug–DNA interactions. Accordingly, ParE and ParC variants predicted to be PPI-neutral were evaluated further using protein stability, evolutionary conservation, and docking analyses to assess non-interface–mediated functional effects.

The GyrA G214V mutation demonstrated a ΔΔG of − 0.60 kcal/mol, exceeding the − 0.5 kcal/mol threshold for significant destabilization and indicating potential disruption of DNA gyrase complex stability, consistent with predictions of impaired catalytic function.

For ribosomal protein L22 (RpLV), mutations exhibited two distinct interaction patterns based on proximity to the interface. Variants located distal to the interface (V71S, I74N, V76R, D77R, G79R, and K83A; mean distance 16.11 Å) produced slight but consistent reductions in binding affinity (ΔΔG = − 0.02 kcal/mol), suggesting minor weakening of ribosomal subunit interactions. In contrast, substitutions positioned closer to the interface (S81E, E78R, and M82H; mean distance 8.11 Å) were associated with increased binding affinity (ΔΔG = + 0.24 kcal/mol), potentially reflecting compensatory structural adaptations that locally enhance ribosomal interactions. Overall, mCSM-PPI predictions indicate that most mutations either weaken or preserve protein–protein interactions, while a subset—particularly within RpLV—exhibits localized increases in inter-subunit binding affinity (Table 5).

**Table 5:** Predicted effects of missense mutations on protein–protein interaction affinity assessed using mCSM-PPI.

Protein	Mutation	mmCSM-PPI ΔΔGBinding
RpoB	D516N	Decrease affinity − 0.54
	H526N	Decrease affinity − 0.27
ParE	D250G	Decrease affinity 0.00
	S458A	Decrease affinity 0.00
ParC	L440R	Decrease affinity 0.00
	S80I	Decrease affinity 0.00
	P577L	Decrease affinity 0.00
	E84G	Decrease affinity 0.00
GyrA	G214V	Decrease affinity − 0.6
Ribosomal protein L22 (rplV)	V71S	Average distance A 16.11 Decrease affinity − 0.02
	I74N	Average distance A 16.11 Decrease affinity − 0.02
	V76R	Average distance A 16.11 Decrease affinity − 0.02
	D77R	Average distance A 16.11 Decrease affinity − 0.02
	G79R	Average distance A 16.11 Decrease affinity − 0.02
	K83A	Average distance A 16.11 Decrease affinity − 0.02
	M82H	Average distance A 8.11 Increase affinity 0.24
	S81E	Average distance A 8.11 Increase affinity 0.24
	E78R	Average distance A 8.11 Increase affinity 0.24

### Analysis of mutational effects on protein stability and molecular flexibility

The structural consequences of resistance-associated missense mutations on protein thermodynamic stability and molecular flexibility were assessed using predicted changes in Gibbs free energy (ΔΔG) and normal mode analysis–derived vibrational entropy changes (ENCoM ΔΔSVib). Overall, most mutations were predicted to exert destabilizing effects, with several also inducing changes in molecular flexibility that may influence protein function and antibiotic interactions.

Both rpoB substitutions (D516N and H526N) were predicted to destabilize the RNA polymerase β subunit. Notably, H526N exhibited stronger destabilization (ΔΔG = − 1.434 kcal/mol) accompanied by increased molecular flexibility (ΔΔSVib = + 0.652 kcal/mol K), suggesting potential alterations in RNA polymerase dynamics that could influence rifampicin binding and transcriptional activity. Similarly, the parE D250G and parC E84G substitutions were associated with destabilization and increased flexibility, changes that may compromise the structural integrity and catalytic function of DNA topoisomerase IV, a key fluoroquinolone target.

The gyrA G214V mutation was predicted to cause moderate destabilization combined with reduced flexibility, indicating impaired conformational adaptability that could affect DNA supercoiling or catalytic efficiency without necessarily abolishing ligand binding. Among ribosomal protein L22 (RpLV) variants, V71S, I74N, and V76R were largely destabilizing and associated with increased flexibility, whereas the G79R substitution uniquely resulted in protein stabilization together with a marked decrease in flexibility (ΔΔSVib = − 1.882 kcal/mol K). This rigidification near the ribosomal exit tunnel may influence macrolide accommodation and ribosomal function. In addition, certain substitutions, including parC S80I and RpLV S81E, exhibited stabilizing effects despite mildly destabilizing ENCoM ΔΔG values, suggesting localized stabilization accompanied by altered dynamic behavior. Missense mutations were identified across multiple antibiotic targets, including GyrA, ParC, ParE, and RpLV, and were associated with variable predictions of protein stability, conformational flexibility, and interaction properties (Table 6).

**Table 6:** Predicted effects of missense mutations on protein stability and molecular flexibility assessed using dynamut and ENCoM.

Protein	Mutation	DynaMut Prediction outcome (ΔΔG kcal/mol)	NMA Based Predictions ENCoM (ΔΔG kcal/mol)	ENCoM (ΔΔSVib kcal mol^− 1^ K^− 1^)
RpoB	D516N	0.109 (Destabilizing)	− 0.225 (Destabilizing)	0.281 (Increase of molecule flexibility)
	H526N	− 1.434 (Destabilizing)	0.522 (Destabilizing)	0.652 (Increase of molecule flexibility)
ParE	D250G	− 0.008 (Destabilizing)	− 0.244 (Destabilizing)	0.306 (Increase of molecule flexibility)
	S458A	1.001 (Stabilizing)	0.075 (Destabilizing)	− 0.094 (Decrease of molecule flexibility)
ParC	L440R	− 0.330 (Destabilizing)	0.134 (Destabilizing)	− 0.168 (Decrease of molecule flexibility)
	S80I	0.271 (Stabilizing)	− 0.058 (Destabilizing)	0.072 (Increase of molecule flexibility)
	P577L	0.250 (Stabilizing)	0.005 (Destabilizing)	− 0.006 (Decrease of molecule flexibility)
	E84G	− 0.741 (Destabilizing)	− 0.362 (Destabilizing)	0.453 (Increase of molecule flexibility)
GyrA	G214V	− 0.665 (Destabilizing)	0.473 (Destabilizing)	− 0.591 (Decrease of molecule flexibility)
Ribosomal protein L22 (rplV)	V71S	− 3.282 (Destabilizing)	− 0.418 (Destabilizing)	0.523 (Increase of molecule flexibility)
	I74N	− 0.693 (Destabilizing)	− 0.323 (Destabilizing)	0.404 (Increase of molecule flexibility)
	V76R	− 0.873 (Destabilizing)	− 0.194 (Destabilizing)	0.243 (Increase of molecule flexibility)
	D77R	0.378 (Stabilizing)	− 0.059 (Destabilizing)	0.074 (Increase of molecule flexibility)
	G79R	0.897 (Stabilizing)	1.506 (Stabilizing)	− 1.882 (Decrease of molecule flexibility)
	K83A	− 0.210 (Destabilizing)	0.030 (Destabilizing)	− 0.037 (Decrease of molecule flexibility)
	M82H	− 0.162 (Destabilizing)	− 0.048 (Destabilizing)	0.060 (Increase of molecule flexibility)
	S81E	0.637 (Stabilizing)	0.243 (Destabilizing)	− 0.303 (Decrease of molecule flexibility)
	E78R	− 0.300 (Destabilizing)	− 0.314 (Destabilizing)	0.392 (Increase of molecule flexibility)

### Molecular docking analysis of antibiotic binding to resistance-associated proteins

#### Docking analysis of CBR-type inhibitor binding to RpoB

**Fig. 2:**
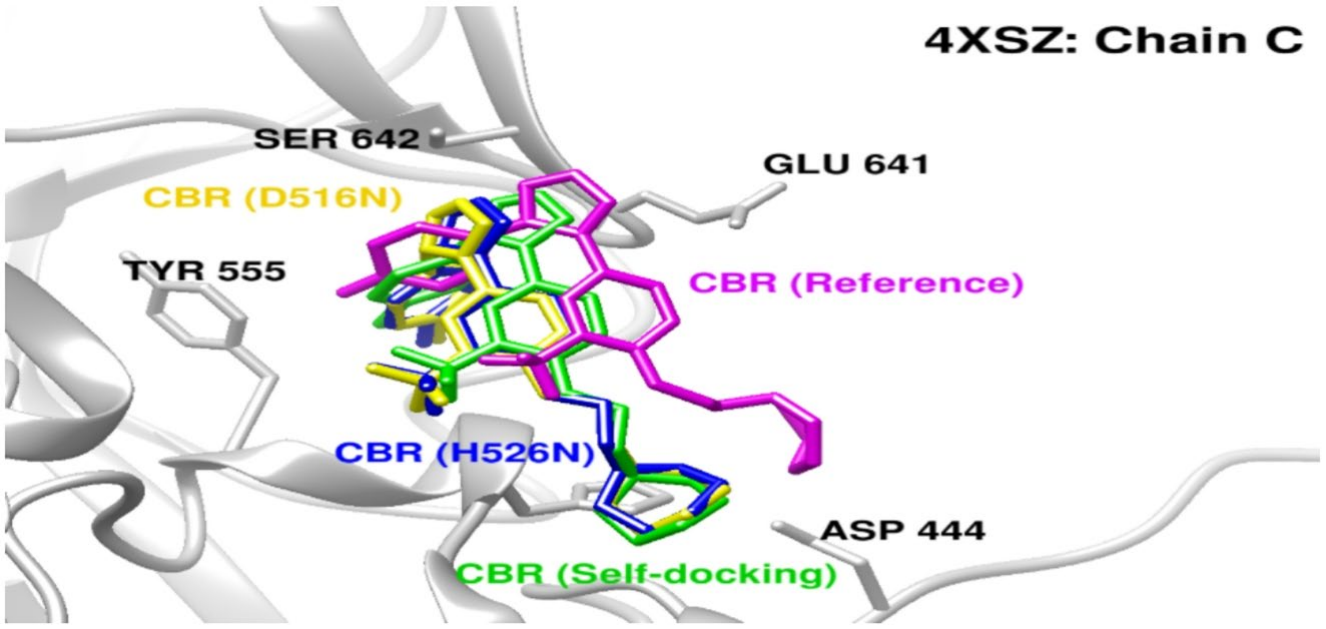
Docking of a CBR-type inhibitor to wild-type and mutant RpoB. Overlay of docking poses shows that the CBR-type inhibitor binds to a similar pocket in wild-type RpoB and in the D516N and H526N mutants, with minimal changes in predicted binding orientation or affinity, indicating preservation of the modeled interaction despite canonical rifampicin resistance mutations.

Molecular docking of a CBR-type inhibitor into the wild-type and mutant forms of *E. coli* RpoB revealed consistently high binding affinity with minimal structural perturbation. In the wild-type model, the inhibitor was positioned deep within the RNA polymerase β-subunit channel, overlapping the established rifampicin-binding pocket (Fig. 2). The predicted binding energy of the wild-type complex was − 7.0 kcal/mol, indicating a stable interaction.

Docking simulations of the D516N and H526N mutant models produced nearly identical binding poses and comparable binding energies (− 6.9 kcal/mol for both mutants), corresponding to a negligible reduction of 0.1 kcal/mol relative to the wild type. In both mutants, the inhibitor remained in the same binding pocket, with only minor local side-chain rearrangements. The D516N substitution replaced a negatively charged aspartate with a neutral asparagine, eliminating the negative charge while preserving hydrogen-bonding capacity. Similarly, the H526N substitution removed the imidazole side chain and associated positive charge but did not alter pocket geometry or ligand accommodation.

Under the docking conditions tested, classical rpoB mutations associated with rifampicin resistance did not produce substantial changes in predicted binding of the CBR-type inhibitor.

#### Docking analysis of Novobiocin binding to ParE

Structural superposition using COOT secondary structure matching aligned the *E.coli* ParE homology model with the Mycobacterium tuberculosis ParE structure (PDB ID: 4URN), enabling identification of a conserved ligand-binding site. Novobiocin coordinates from the 4URN complex were used to define the docking region, and simulations were performed using AutoDock4.

**Fig. 3:**
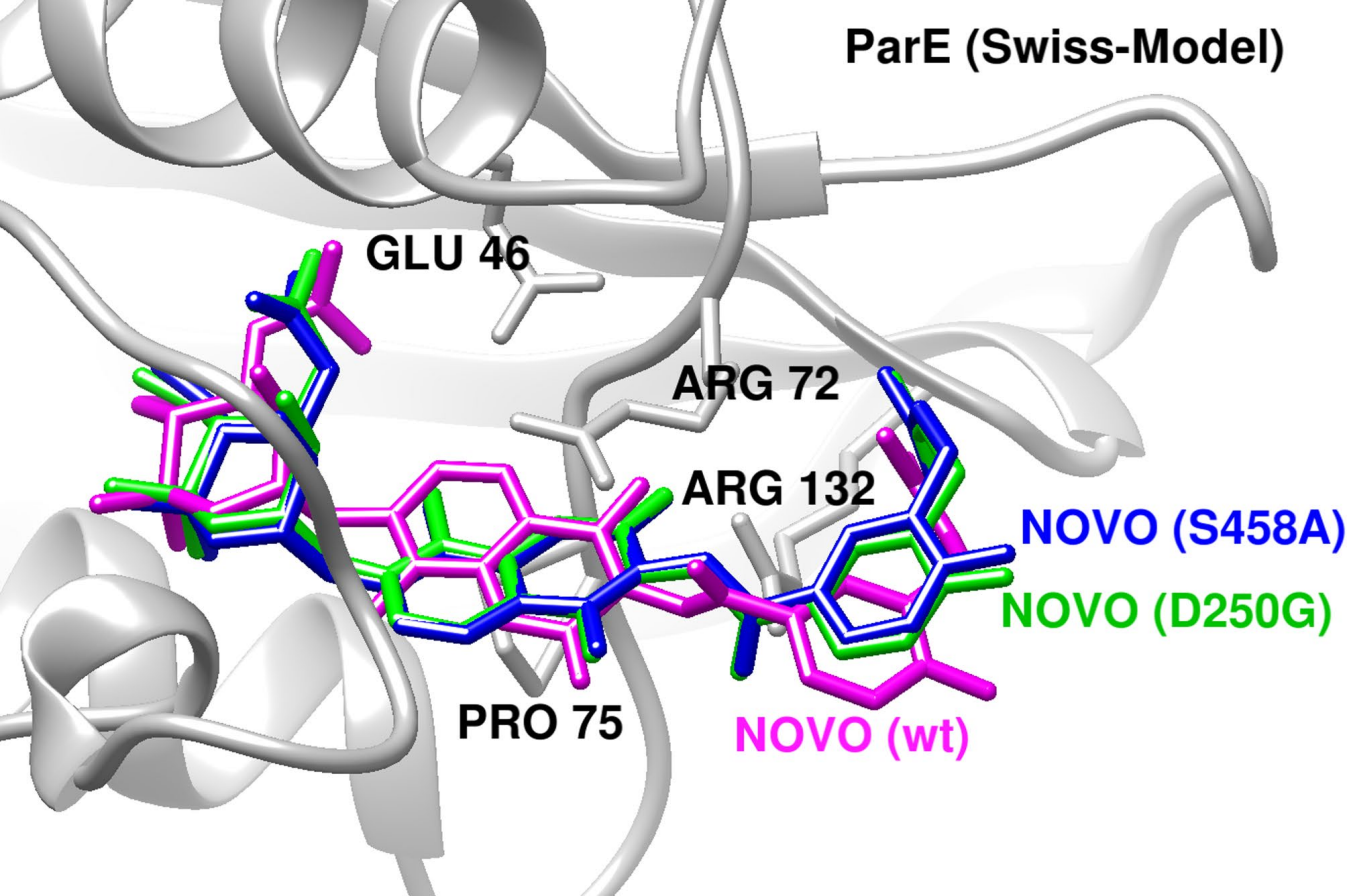
Novobiocin binding to ParE and resistance-associated mutants. Docking poses illustrate that novobiocin adopts a stable conformation in wild-type ParE, while the D250G and S458A substitutions alter ligand positioning and reduce predicted binding, suggesting weakened modeled interactions.

In the wild-type ParE model, novobiocin adopted a stable binding pose within the predicted pocket, with a binding energy of − 9.2 kcal/mol. Docking into the D250G and S458A mutant models resulted in reduced binding affinities of − 8.1 and − 8.0 kcal/mol, respectively. These reductions were observed as decreases in predicted docking scores relative to the wild-type complexes. (Fig. 3).

#### Docking analysis of Trovafloxacin binding to parc

Protein–DNA docking using HDOCK was performed to evaluate trovafloxacin binding to the *E. coli* ParC subunit of topoisomerase IV. The inhibitor localized at the protein–DNA interface, consistent with its established interfacial inhibition mechanism. Redocking into the wild-type ParC–DNA complex (PDB ID: 4Z53) yielded a high docking score (− 295.26), reproducing the crystallographic pose.

**Fig. 4:**
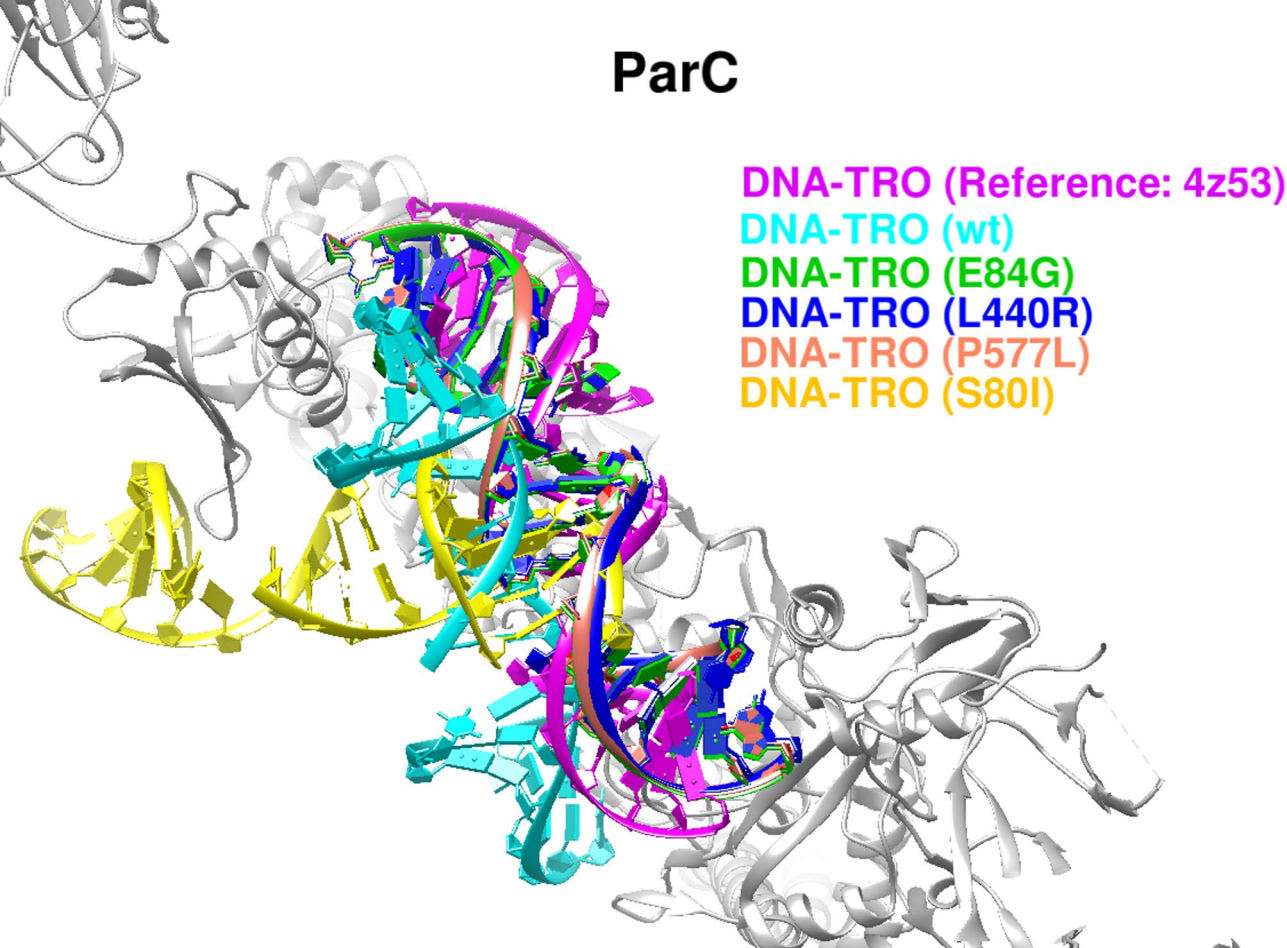
Trovafloxacin docking to ParC and resistance-associated mutants. Superposition of DNA–trovafloxacin complexes shows that the S80I and E84G mutations markedly alter predicted drug binding relative to the wild type, whereas L440R and P577L produce minimal changes, indicating mutation-specific effects on modeled fluoroquinolone interactions.

Mutation analysis revealed variable effects on trovafloxacin binding. The L440R substitution produced a docking score similar to the wild type (− 294.43), indicating minimal disruption of drug binding. In contrast, the S80I and E84G substitutions showed substantially reduced docking scores (− 221.13 and − 212.18, respectively), consistent with impaired drug accommodation at the cleavage complex. The P577L mutation resulted in a moderately reduced score (− 281.38), suggesting partial retention of binding capacity (Fig. 4).

#### Docking analysis of Moxifloxacin binding to GyrA

Because the available *E.coli* GyrA crystal structure (PDB ID: 4Z2C) was incomplete, a full-length homology model was generated using SWISS-MODEL. Protein–DNA docking with moxifloxacin was performed using HDOCK.

**Fig. 5:**
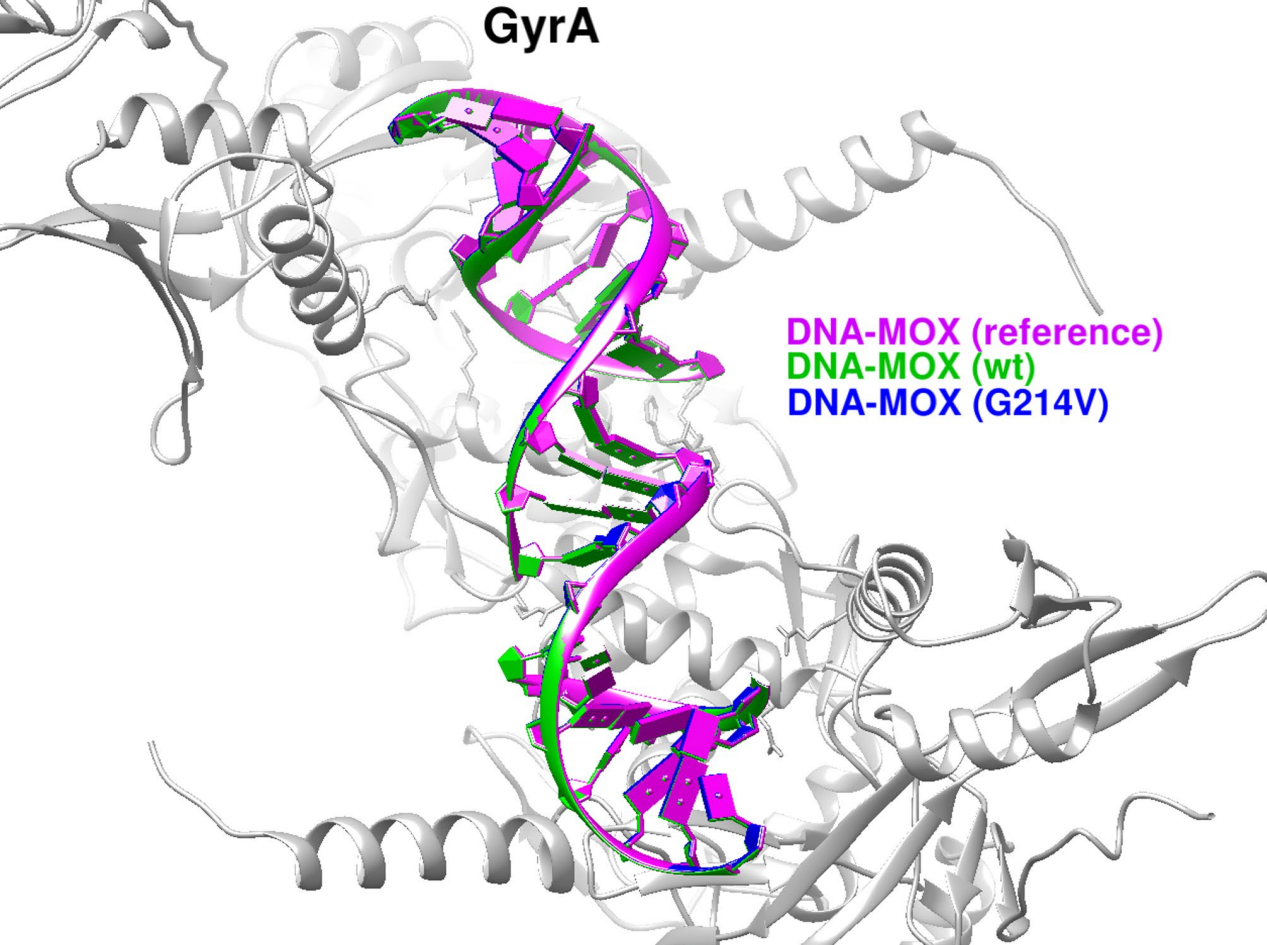
Moxifloxacin docking to GyrA and the G214V mutant. Superposition of DNA–moxifloxacin complexes shows that the G214V substitution does not substantially alter the predicted binding pose or orientation relative to the wild-type GyrA model, indicating preservation of modeled fluoroquinolone binding despite the mutation.

The wild-type GyrA–DNA–moxifloxacin complex exhibited a strong docking score of − 541.01. The G214V mutant showed a comparable score (− 542.73), with no appreciable displacement or reorientation of the ligand (Fig. 5). Under the docking conditions tested, the G214V substitution did not produce a marked change in predicted moxifloxacin binding, despite being predicted to destabilize protein structure in stability analyses.

#### Docking analysis of erythromycin binding to RpLV (ribosomal protein L22)

**Fig. 6:**
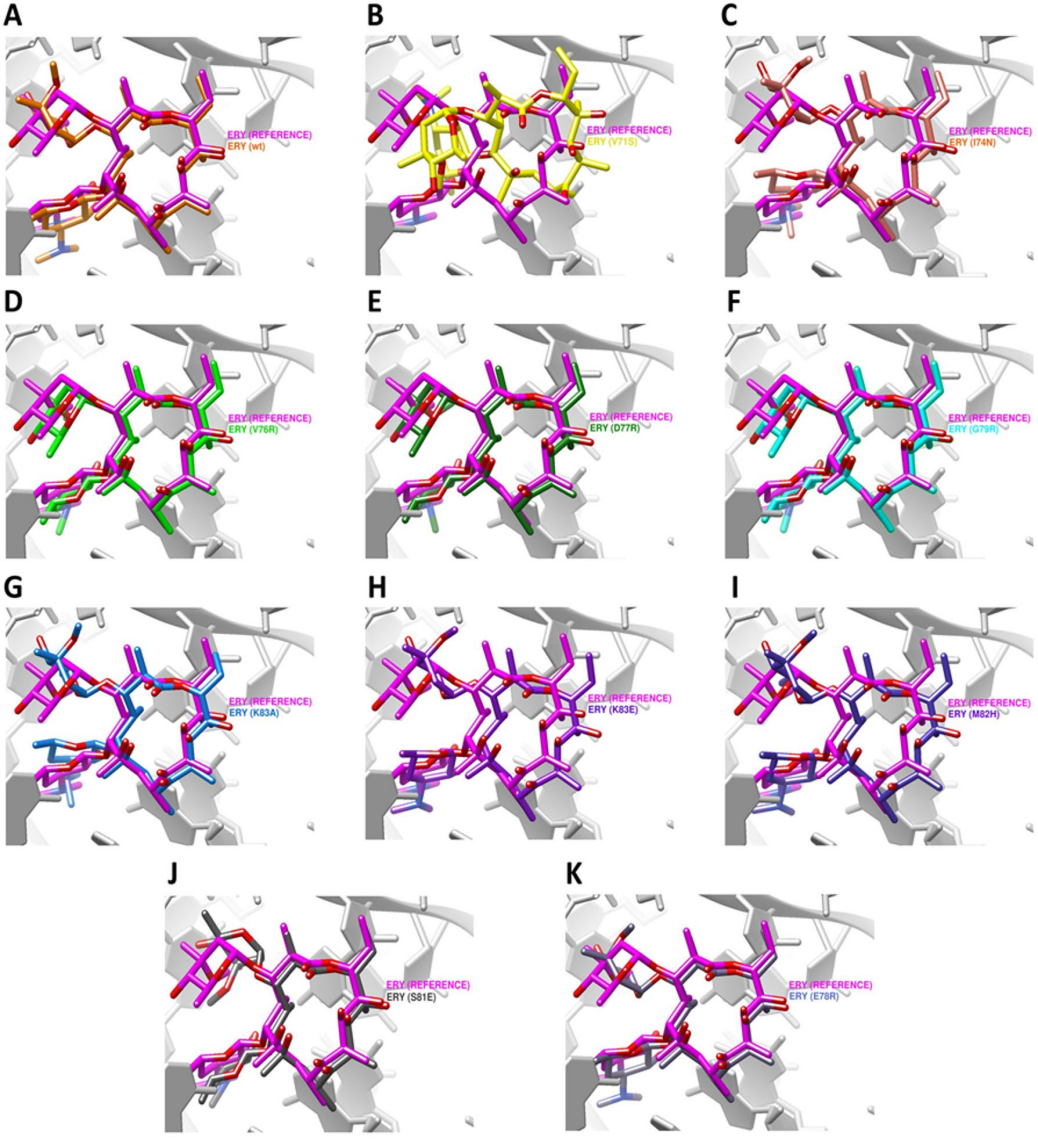
Erythromycin docking to wild-type and mutant ribosomal protein L22 (rplV). Structural overlays show mutation-specific differences in predicted erythromycin binding poses within the macrolide tunnel, with several ribosomal protein L22 (rplV). substitutions altering ligand orientation relative to the wild type, while others retain poses similar to the reference, indicating heterogeneous modeled effects on macrolide interaction.

Docking of erythromycin (ERY) to wild-type and mutant RpLV models was performed using a focused docking region centered on the macrolide-binding tunnel. Self-docking into the reference structure produced a baseline binding energy of − 5.7 kcal/mol, supporting the validity of the docking protocol. Docking into mutant RpLV variants revealed heterogeneous effects on ERY binding. Moderate reductions in predicted binding affinity were observed for V71S (− 4.7 kcal/mol), I74N (− 5.1 kcal/mol), and K83A (− 4.9 kcal/mol), whereas more pronounced reductions were detected for V76R, D77R, and G79R (approximately − 4.0 to − 4.1 kcal/mol), consistent with disruption of interactions within the macrolide-binding tunnel. In contrast, several substitutions retained binding energies comparable to the wild type, including K83E (− 5.4 kcal/mol), M82H (− 5.2 kcal/mol), and S81E (− 5.5 kcal/mol), while the E78R variant exhibited the most favorable predicted binding energy (− 5.8 kcal/mol) (Fig. 6). Collectively, ribosomal protein L22 (rplV).substitutions exhibited variable, mutation-specific effects on predicted erythromycin binding under the docking conditions tested.

#### Molecular docking analysis of *E. coli* proteins

Molecular docking analyses showed that the effects of resistance-associated mutations on antibiotic binding affinity varied across target proteins. For RpoB, the canonical resistance mutations D516N and H526N produced negligible changes in binding to the CBR-type inhibitor (− 6.9 kcal/mol versus − 7.0 kcal/mol for the wild type), indicating preservation of drug–target interactions. In contrast, ParE variants D250G and S458A exhibited reduced binding to novobiocin (− 8.5 kcal/mol versus − 9.2 kcal/mol), reflecting a modest but consistent decrease in affinity. Among ParC variants, the canonical S80I and the novel E84G mutations resulted in substantial reductions in trovafloxacin binding (− 221.13 and − 212.18, respectively, compared with − 295.26 for the wild type), whereas L440R and P577L showed only minor effects. For GyrA, the novel G214V variant retained binding affinity to moxifloxacin (− 542.73 versus − 541.01), indicating minimal impact on predicted ligand binding. In contrast, multiple ribosomal protein L22 (rplV). substitutions identified in isolate UMX99540.1 weakened erythromycin binding relative to the wild type (− 4.0 to − 5.5 kcal/mol versus − 5.7 kcal/mol), with the largest reductions observed for D77R (− 4.0 kcal/mol) and V76R (− 4.1 kcal/mol).

Overall, these results indicate that while RpoB resistance-associated mutations preserve antibiotic binding, several mutations in ParE, ParC, and ribosomal protein L22 (rplV). are associated with reduced ligand affinities, providing a structural framework for interpreting resistance-associated variation observed in the analyzed isolates (Table 7). The structural figures and docking comparisons presented in this study illustrate relative conformational and interaction differences between wild-type and mutant models. These visual analyses are intended to support qualitative, hypothesis-generating interpretations rather than to provide quantitative predictions of antimicrobial resistance phenotypes.

**Table 7:** Comparative molecular docking analysis of wild-type and mutant *E. coli* proteins with their respective inhibitors.

Protein	Number of strains	Mutation	Ligand	Binding affinity score	
				AD4	HDock
RpoB	–	wt	CBR type inhibitor	− 7.0	
	3	D516N	CBR type inhibitor	− 6.9	
	1	H526N	CBR type inhibitor	− 6.9	
ParE	–	wt	Novobiocin	− 9.2	
	1	D250G	Novobiocin	− 8.5	
	9	S458A	Novobiocin	− 8.5	
ParC	–	wt	DNA-Trovafloxacin		− 295.26
	1	L440R	DNA-Trovafloxacin		− 294.43
	20	S80I	DNA-Trovafloxacin		− 221.13
	1	P577L	DNA-Trovafloxacin		− 281.38
	1	E84G	DNA-Trovafloxacin		− 212.18
GyrA	-	wt	DNA-Moxifloxacin		− 541.01
	1	G214V	DNA-Moxifloxacin		− 542.73
Ribosomal protein L22 (rplV).	–	wt	Erythromycin	− 5.7	
	(One strain) UMX99540.1	V71S	Erythromycin	− 4.7	
		I74N	Erythromycin	− 5.1	
		V76R	Erythromycin	− 4.1	
		D77R	Erythromycin	− 4.0	
		G79R	Erythromycin	− 4.1	
		K83A	Erythromycin	− 4.9	
		K83E	Erythromycin	− 5.4	
		M82H	Erythromycin	− 5.2	
		S81E	Erythromycin	− 5.5	
		E78R	Erythromycin	− 5.5	

*For interpretation, docking differences of ≥ 1.0 kcal/mol (AutoDock ΔG) or ≥ 50 HDOCK score units were classified as substantial, whereas smaller shifts were considered minor and interpreted cautiously in light of potential stochastic variation.

## Discussion

Antimicrobial resistance in *E.coli* remains a major global public health challenge, particularly in resource-limited settings such as Sudan, where therapeutic options are constrained and surveillance capacity remains limited^23^. This study provides the first comprehensive structural bioinformatics characterization of resistance-associated missense mutations in Sudanese *E.coli* clinical isolates. By extending beyond canonical mechanisms of fluoroquinolone, macrolide, and rifampicin resistance, our analysis reveals a substantial burden of novel variants with potential functional and clinical relevance. Importantly, the docking analyses presented here are intended to generate mechanistic hypotheses regarding potential effects of missense mutations on drug–target interactions, rather than to serve as direct predictors of antimicrobial resistance in the absence of experimental validation.

A central conceptual framework of this study is the distinction between canonical and non-canonical resistance-associated mutations. Canonical mutations, defined as previously reported substitutions with established links to antimicrobial resistance, served as internal benchmarks to validate the analytical pipeline. In contrast, non-canonical mutations comprising novel or infrequently reported variants—were emphasized for their potential to reveal resistance mechanisms not captured by current surveillance paradigms. This distinction guided interpretation rather than analytical inclusion: both mutation classes were subjected to the same computational analyses, while non-canonical variants were prioritized in comparative structural, stability, and docking analyses to assess their potential functional relevance beyond known resistance pathways.

The distribution of missense mutations across resistance-associated proteins observed in this study is consistent with patterns reported in previous whole-genome sequencing studies of E.coli^38^.

In particular, the predominance of mutations in gyrA and parC reflects their well-established role as primary chromosomal targets of fluoroquinolone resistance, as widely documented in clinical isolates from diverse geographic regions^39^. Similar mutation distributions, characterized by frequent substitutions in QRDR-associated genes and lower mutation frequencies in gyrB and parE, have been reported in studies from Africa, Asia, and Europe^40^.

The relatively low number of substitutions detected in rpoB and rpoC is also in agreement with prior observations indicating that rifampicin resistance–associated mutations occur less frequently in *E. coli* than fluoroquinolone-associated substitutions and are often restricted to specific selective contexts^41^. Notably, the presence of multiple substitutions in ribosomal protein L22 (rplV) parallels earlier reports describing L22 as a secondary target of macrolide-associated resistance (43.). However, the high proportion of previously unreported L22 variants observed in this study appears greater than that described in most existing datasets, suggesting potential regional enrichment of non-canonical variants.

Together, these findings indicate that the mutation numbers and distributions shown in Fig. 1 conform to established global resistance trends while also revealing locally distinctive mutational features in Sudanese *E. coli* isolates.

Among the 71 missense mutations identified, 19 variants were classified as deleterious based on consensus pathogenicity predictions, of which 13 (68.4%) were novel. This proportion of deleterious variants is comparable to previous computational analyses of *E.coli* genomes, which consistently report that only a subset of detected missense mutations are predicted to have strong functional impact, with the majority being neutral or weakly deleterious^43^. Notably, ribosomal protein L22 (rplV). harbored the largest cluster of damaging substitutions, with all such variants detected within a single isolate (UMX99540.1). While ribosomal protein L22 has previously been implicated as a secondary target of macrolide-associated resistance, most published datasets are dominated by a limited number of recurrent mutations rather than extensive clusters of novel variants^42^. In contrast, deleterious canonical substitutions were confined to rpoB (D516N and H526N), parE (D250G and S458A), and parC (E84G and S80I), all of which are well-established resistance-associated targets and are frequently classified as functionally damaging in both experimental and in silico studies^41^. The predominance of novel deleterious variants observed here therefore exceeds what is typically reported in global *E. coli* surveillance studies and suggests the presence of resistance-associated mutational patterns that are not yet captured by current reference databases. This finding underscores the importance of integrating locally derived genomic data with structural and functional analyses to improve diagnostic accuracy and resistance monitoring in underrepresented regions.

In the context of rifampicin resistance, substitutions in rpoB within the rifampicin resistance–determining region (D516N, H526N, and S531L) reaffirm their established role in altering RNA polymerase function and reducing drug susceptibility^44,45^. The concurrent presence of compensatory mutations in rpoC (e.g., Q1126K and L731I) likely mitigates the associated fitness costs, thereby facilitating the persistence of resistant strains. Consistent with this interpretation, molecular docking analyses demonstrated that CBR-type inhibitors retained binding to alternative polymerase sites, suggesting potential therapeutic relevance in contexts where rifampicin efficacy is compromised.

For fluoroquinolones, several rare and non-canonical variants expanded the resistance landscape observed in Sudanese isolates. The parE D250G substitution was predicted to reduce novobiocin affinity through second-shell destabilization, whereas gyrA G214V preserved moxifloxacin binding despite consistent destabilization of the gyrase complex. This apparent dissociation between ligand binding and protein stability highlights that resistance-associated mutations are not necessarily mediated solely through impaired drug–target interactions. Instead, preserved ligand binding in the presence of destabilization or altered dynamics may reflect compromised enzymatic efficiency, altered conformational flexibility, or reduced stability of the drug–target complex under physiological conditions, thereby creating selective pressure for compensatory adaptations. Comparable destabilizing yet resistance-permissive substitutions have been reported in Mycobacterium tuberculosis (rpoB, katG)^44,46^ and in *E. coli* folA mutants^47^.

Notably, most ParE and ParC substitutions were predicted to be protein–protein interaction (PPI) neutral, indicating that these variants are unlikely to disrupt topoisomerase IV subunit assembly. Rather, their functional impact appears to be mediated primarily through local destabilization, altered conformational dynamics, or impaired drug–DNA interactions, consistent with the observed reductions in structural stability and fluoroquinolone docking scores^48,49^. Within this unified interpretive framework, reduced predicted binding affinity is interpreted as one potential mechanism of resistance, whereas preserved docking scores do not imply functional neutrality. Instead, docking outcomes are interpreted in conjunction with pathogenicity predictions, stability changes, evolutionary conservation, and structural localization. This integrative approach allows discrimination between mutations that primarily affect ligand binding and those that modulate resistance through indirect structural or functional mechanisms, reconciling apparently divergent docking outcomes and supporting a coherent, biologically plausible interpretation of resistance-associated variation^46,50^. A particularly notable finding of this study was the clustering of macrolide resistance–associated substitutions in ribosomal protein L22 (rplV), localized within the β-hairpin loop lining the nascent peptide exit tunnel. Several variants, including V76R, D77R, and G79R, were predicted to disrupt erythromycin binding, likely through steric interference within the tunnel, whereas others (e.g., M82H and K83E) appeared to preserve tunnel flexibility, reflecting structural adaptability under antibiotic pressure^40,51^. Given the frequent empirical use of macrolides in Sudan, mutational heterogeneity within ribosomal protein L22 (rplV). may contribute to variability in macrolide interaction at the structural level.

Importantly, all ribosomal protein L22 (rplV). missense mutations identified in this study were confined to a single isolate and should therefore be interpreted as an isolate-specific phenomenon rather than evidence of a broader population-level trend. This distinction is particularly relevant for ribosomal proteins, where antibiotic binding is governed by complex interactions involving rRNA, neighboring ribosomal proteins, and the conformational dynamics of the exit tunnel. Consequently, the docking results for L22 variants should be interpreted as structural hypotheses rather than direct predictors of macrolide resistance, especially given that the analyses were performed using isolated protein models rather than complete ribosomal assemblies. While the observed clustering highlights the potential for localized accumulation of resistance-associated variation, its broader epidemiological significance will require validation in larger and more diverse strain collections.

Neutral polymorphisms—particularly those observed in gyrB and rpoC—highlight the importance of accurate variant classification to avoid false-positive resistance predictions in diagnostic settings^52^. Misclassification could distort epidemiological estimates and compromise antimicrobial stewardship efforts. Furthermore, the absence of chromosomal missense mutations in plasmid-associated resistance genes emphasizes the need for complementary plasmid-focused and transcriptomic analyses to fully characterize the resistance repertoire of Sudanese isolates^53,54^.

Comparisons with other regions reveal notable parallels. In Nigeria and Ethiopia, *E. coli* isolates exhibit fluoroquinolone and β-lactam resistance rates exceeding 70%, largely driven by QRDR mutations and plasmid-mediated determinants^55,56^. Similarly, studies from India and Pakistan report widespread QRDR substitutions and ribosomal protein alterations, reflecting convergent evolutionary trajectories under sustained antibiotic pressure^55,57^. Such convergence—where identical or functionally equivalent mutations (e.g., gyrA S83L and parC S80I) arise independently—underscores predictable patterns of antimicrobial resistance evolution in high-burden settings.

At the same time, plasmid-mediated resistance displays marked regional variability. Genes such as qnrB and qnrS are highly prevalent in South Asia, frequently co-localized with ESBLs and carbapenemases, whereas chromosomal QRDR substitutions remain dominant in sub-Saharan Africa^58,59^. Although plasmid-level variants were not captured in the present study, this limitation highlights the importance of integrated genomic surveillance approaches that encompass both chromosomal and mobile resistance determinants to track cross-border transmission. Moreover, rpoB mutations have been shown to promote bacterial persistence through pleiotropic fitness effects under antibiotic pressure^60^, further supporting the role of fitness-restoring adaptations in sustaining resistant lineages across LMICs. Collectively, these observations reinforce the value of harmonized initiatives such as WHO GLASS and the Africa CDC Pathogen Genomics Initiative.

In summary, this study demonstrates a predominance of novel resistance-associated variants in Sudanese *E. coli*, with clusters in ribosomal protein L22 (rplV). and rare substitutions in parE and gyrA expanding the known mutational landscape beyond classical markers. Clinically, these findings highlight the need for diagnostics tailored to local mutational profiles. Epidemiologically, they place Sudan’s resistance patterns within broader LMIC trends while revealing region-specific pathways. Finally, the identification of alternative binding sites and emerging resistance-associated mutations underscores opportunities for rational drug design and targeted surveillance, emphasizing the value of integrated genomic and structural approaches in addressing the global antimicrobial resistance crisis.

### Limitations of the study

#### Lack of experimental validation

Although this study provides strong in silico predictions regarding the structural and functional consequences of missense mutations, the absence of in vitro or in vivo experiments limits confirmation of their biochemical and phenotypic impacts.

#### Limited geographic and strain diversity

The analysis was restricted to *E. coli* strains isolated from Sudan. While these data yield important insights into local resistance patterns, the findings may not be fully representative of global *E. coli* populations with broader genetic diversity.

#### Focus limited to missense mutations

This study investigated only missense mutations within protein-coding regions. Other resistance mechanisms—such as regulatory mutations, gene amplifications, efflux pump alterations, and horizontal gene transfer—were not considered.

#### Exclusion of plasmid-mediated determinants

Chromosomal mutations were the primary focus of this work. Plasmid-mediated resistance determinants, including extended-spectrum β-lactamases (ESBLs), carbapenemases, and plasmid-mediated quinolone resistance (PMQR) genes, were not analyzed, despite their well-established role in multidrug resistance. Future studies integrating plasmid-resolved sequencing are needed to provide a more comprehensive view of the resistance landscape.

### Docking simplifications

Docking analyses, particularly for ribosomal protein L22 (rplV), were conducted using simplified protein-based models rather than complete ribosome–RNA assemblies. While this approach enabled relative comparisons of wild-type and mutant variants, it does not fully replicate the native structural context in which antibiotic binding occurs. Therefore, the docking results should be interpreted as indicative rather than definitive, pending validation in more biologically representative models.

## Conclusion

This study presents the first structural bioinformatics analysis of resistance-associated missense mutations in Sudanese *E. coli*, revealing a predominance of previously unreported (novel) variants alongside a smaller number of well-characterized canonical mutations. Several non-classical substitutions, including GyrA-G214V and ParE-D250G, illustrate how novel mutations can destabilize target proteins without completely abolishing drug binding, while clustered novel variants in ribosomal protein L22 (rplV) provide a structural explanation for heterogeneous macrolide responses. In contrast, classical rpoB resistance mutations remained compatible with binding of CBR-type inhibitors, highlighting potential therapeutic opportunities despite rifampicin resistance. Collectively, these findings expand the known mutational landscape of antimicrobial resistance in *E. coli* and demonstrate the value of in silico structural approaches for identifying emerging resistance mechanisms in settings where experimental validation is constrained. Future studies integrating experimental confirmation with plasmid-resolved and transcriptomic sequencing will be essential to translate these insights into improved diagnostics, antimicrobial stewardship, and therapeutic innovation in high-burden regions.

## Materials and methods

### Overview of the in silico analysis

In this study, we employed a comprehensive in silico framework integrating multiple bioinformatics tools to analyze missense genetic variants associated with antimicrobial resistance. The workflow encompassed prediction of variant pathogenicity, assessment of mutation-induced changes in protein thermodynamic stability, evaluation of evolutionary conservation, comparison of wild-type and mutant protein structures, analysis of protein–ligand interactions through molecular docking, and investigation of protein–protein interaction effects. This multi-step strategy was designed to prioritize mutations with potential functional relevance while ensuring methodological consistency and reproducibility.

To resolve conflicting predictions generated by individual computational tools, a predefined hierarchical integration strategy was applied. For SNP pathogenicity assessment, variants were classified as deleterious only when a majority consensus (≥ 4 out of 6 predictors) was achieved using PredictSNP; variants failing to meet this threshold were classified as neutral or uncertain and were not prioritized for downstream analyses.

For protein stability assessment, predictions from five independent tools (mCSM, I-Mutant2.0, SDM, DUET, and MuPro) were integrated, and a mutation was considered stabilizing or destabilizing only when at least three tools concurred on the direction of the predicted ΔΔG change. In cases of disagreement, consensus directionality rather than absolute ΔΔG magnitude was prioritized.

When pathogenicity or stability predictions remained ambiguous, additional complementary criteria were applied, including evolutionary conservation (ConSurf score ≥ 6), functional impact prediction (MutPred2 score ≥ 0.5), and structural context, such as localization within conserved domains or proximity to known functional or ligand-binding sites. Only variants supported by multiple independent lines of evidence were advanced to molecular docking, protein–protein interaction analysis, and dynamic stability assessment. This integrative, multi-criterion prioritization framework minimizes tool-specific bias and supports biologically plausible interpretation of resistance-associated mutations.

Canonical mutations were defined as previously reported resistance-associated substitutions with established phenotypic or mechanistic evidence in global databases and published literature. In contrast, non-canonical mutations comprised variants that were absent from Sudanese datasets and/or infrequently reported in global surveillance databases and lacked prior functional or structural characterization. This distinction informed interpretation rather than analytical inclusion: both canonical and non-canonical variants were subjected to the same computational pipeline, while non-canonical variants were prioritized for structural, stability, and docking analyses to assess their potential functional relevance beyond known resistance mechanisms.

### Data retrieval

Proteins associated with antibiotic resistance mechanisms—including quinolone resistance–determining region (QRDR) proteins (gyrA, gyrB, parC, and parE), ribosomal proteinr (RpsL, RrplD, ribosomal protein L22 (rplV)), and RNA polymerase subunits (rpoB and rpoC)—were retrieved from whole-genome sequencing data of Escherichia coli strains isolated in Sudan and deposited in the NCBI database (https://www.ncbi.nlm.nih.gov/). Three BioProjects were included: PRJNA806525 (21 isolates), PRJNA526349 (1 isolate), and PRJNA767482 (33 isolates), comprising a total of 55 E. coli genomes.

The E. coli K-12 substrain MG1655 reference genome (RefSeq NC_000913.3; GenBank accession U00096.3) was used for sequence alignment and comparative analyses. Protein-coding sequences were extracted in FASTA format for downstream multiple sequence alignment and variant identification.

### SNP calling and variant classification

Protein-level single nucleotide polymorphisms (SNPs) were identified through multiple sequence alignment. For each resistance-associated protein, a dataset of 66 protein sequences in FASTA format was compiled, comprising the Escherichia coli K-12 substrain MG1655 reference sequence, 10 representative E. coli sequences retrieved from NCBI RefSeq, and 55 sequences from Sudanese isolates. Multiple sequence alignments were performed using Clustal Omega v1.2.4 (https://www.ebi.ac.uk/Tools/msa/clustalo/) with default parameters.

Amino acid substitutions were identified relative to the MG1655 reference sequence, systematically annotated, and retained for downstream analyses, including pathogenicity prediction, protein stability assessment, evolutionary conservation analysis, and structural modeling.

Missense variants were classified as novel when the corresponding amino acid substitutions were absent from publicly available global antimicrobial resistance and variant databases, including NCBI RefSeq annotations and curated resistance repositories, at the time of analysis. Novelty was defined strictly at the sequence level and did not imply the absence of prior functional or structural characterization of the affected protein. Variants previously reported in the literature or databases were classified as canonical, irrespective of their presence or absence in Sudanese datasets.

### SNP pathogenicity analysis

The pathogenicity of missense SNPs was evaluated using PredictSNP 1.0 (https://loschmidt.chemi.muni.cz/predictsnp/), a consensus-based prediction platform^20,21^. PredictSNP integrates six established algorithms: Multivariate Analysis of Protein Polymorphism (MAPP), nsSNPAnalyzer, Protein Analysis through Evolutionary Relationships (PANTHER), PhD-SNP, PolyPhen-1/2, Sorting Intolerant From Tolerant (SIFT), and Synonymous Non-synonymous Analysis Program (SNAP).

For each variant, the number of constituent tools predicting a damaging effect was recorded, and a majority-vote rule was applied: variants with ≥ 4/6 damaging predictions were classified as deleterious; 3/6 were classified as uncertain; and ≤ 2/6 were classified as neutral. PredictSNP normalized confidence scores (%) were reported for transparency but were not used as an additional cutoff. Jobs were re-run if any tool failed, and variants with fewer than six available predictions were excluded from deleterious counts. Raw per-tool outputs, consensus calls, and confidence values were archived from the PredictSNP Summary (CSV) export to enable exact reproducibility^21,26^.

### Prediction of protein stability changes upon mutation

To assess the impact of SNPs on protein stability, five computational tools were used: mCSM, I-Mutant2.0, SDM, DUET, and MuPro^22,27^. These tools predict changes in Gibbs free energy (ΔΔG) following amino acid substitutions, with negative values indicating destabilization and positive values indicating stabilization.

A mutation was classified as stabilizing or destabilizing when at least three of the five tools were in agreement. A ΔΔG cutoff of 0 kcal/mol was applied across tools. The magnitude of ΔΔG was further categorized as strongly destabilizing ( < − 1.0 kcal/mol), moderately destabilizing (− 1.0 to − 0.5 kcal/mol), neutral (− 0.5 to + 0.5 kcal/mol), moderately stabilizing (+ 0.5 to + 1.0 kcal/mol), or strongly stabilizing ( > + 1.0 kcal/mol). Mutations with conflicting predictions were labeled as having an uncertain effect and evaluated further using conservation and structural context.

### Identification and localization of protein mutations

The positional distribution of SNPs within protein sequences was analyzed using InterPro (https://www.ebi.ac.uk/interpro/), which integrates multiple protein signature databases to identify conserved domains, motifs, and functional sites^23,28^. This analysis enabled mapping of mutations relative to critical structural and functional regions.

Prediction of SNP conservation and structural effects.

Evolutionary conservation of amino acid residues was assessed using ConSurf (https://consurf.tau.ac.il/)^24,29^. Residues with ConSurf scores of 8–9 were considered highly conserved, whereas scores of 1–3 indicated variable regions. Structural and functional consequences of mutations were further evaluated using MutFunc^25,30^ and MutPred2^21,31^. MutPred2 scores ≥ 0.5 were considered potentially deleterious, and scores ≥ 0.8 were considered highly confident. Mutations were prioritized when they occurred at conserved positions and/or were predicted to affect structural integrity or molecular interactions.

### Impact of SNPs on protein–protein interactions

The effect of mutations on protein–protein interaction (PPI) binding affinity was predicted using mCSM-PPI (https://biosig.lab.uq.edu.au/mcsm_ppi2/)^26,32^. Changes in binding free energy (ΔΔG_binding) were calculated, with values ≤ − 0.5 kcal/mol considered indicative of significant destabilization of PPIs.

Prediction of mutation effects on protein stability and dynamics.

DynaMut was used to predict mutation-induced changes in protein stability and flexibility by integrating graph-based signatures with normal mode analysis^27,33^. Mutations with ΔΔG ≤ − 0.5 kcal/mol were classified as significantly destabilizing and prioritized for further structural interpretation. DynaMut consensus predictions incorporate ENCoM and DUET outputs via a Random Forest model trained on experimentally validated mutation datasets.

### Docking analysis

Protein structures were obtained from the RCSB Protein Data Bank or generated using SWISS-MODEL^28,34^. Structures were prepared in AutoDockTools by adding polar hydrogens, merging non-polar hydrogens, assigning Gasteiger charges, and converting files to PDBQT format^29^. Binding grids were generated using AutoGridFR^30,35^.

Protein–ligand docking was performed using AutoDock4 with the Lamarckian genetic algorithm, retaining the ten lowest-energy poses for analysis^31,36^. Protein–DNA docking was conducted using HDOCK following generation of wild-type and mutant models in UCSF Chimera via the “swapaa” function^32,37^. Docking scores were interpreted as relative measures of binding affinity.

Note: Changes in docking scores represent relative differences in predicted ligand–target interactions between wild-type and mutant models and do not directly equate to antimicrobial resistance phenotypes, which are influenced by additional factors including enzymatic activity, protein dynamics, cellular context, and drug transport.

### Docking validation and interpretation

Docking protocols were validated using representative crystallographic complexes, where redocking reproduced native ligand-binding poses with root-mean-square deviation (RMSD) values < 2.0 Å when experimental structures were available. For homology-modeled proteins, validation was based on the reproducibility of ligand orientation and docking scores across wild-type and mutant variants.

For ribosomal protein L22 (rplV), erythromycin docking was performed using a simplified protein-based model focused on the macrolide-binding tunnel. Although this approach does not capture full ribosome–rRNA interactions, it enables relative comparison of ligand binding between wild-type and mutant variants. Docking score differences ≥ 1.0 kcal/mol (AutoDock) or ≥ 50 HDOCK score units were considered substantial, whereas smaller differences were interpreted cautiously and evaluated in conjunction with protein stability and evolutionary conservation analyses.

Throughout this study, docking scores were treated as the primary quantitative criterion for interpreting mutation-induced changes in ligand binding. Qualitative inspection of binding poses was used solely as a supportive tool to contextualize quantitative score differences and was not applied independently to infer resistance. Structural pose variations were emphasized only when accompanied by docking score changes meeting or exceeding predefined significance thresholds; cases with marginal score differences were interpreted conservatively to avoid overinterpretation (Table 8).

**Table 8:** Validation of molecular docking protocols used for representative protein–ligand complexes.

Protein	Ligand (control)	PDB ID/model	Validation approach	Result/baseline score
ParC	Trovafloxacin	4Z53	Redocking of co-crystallized ligand	RMSD < 2.0 Å (validated pose)
RpoB	CBR-type inhibitor	4XSZ	Redocking into crystallographic site	ΔG − 7.0 kcal/mol (consistent)
GyrA	Moxifloxacin	SWISS-MODEL (4Z2C template)	Pose reproducibility across wild-type and mutant variants	Stable orientation and binding score
ParE	Novobiocin	SWISS-MODEL (4URN template)	Pose reproducibility across wild-type and mutant variants	Consistent docking energies
Ribosomal protein L22 (rplV)	Erythromycin	Homology/reference model	Self-docking to reference model	ΔG − 5.7 kcal/mol (baseline)

### Use of Language model tools

This manuscript was supported in part by the use of a large language model (ChatGPT, developed by OpenAI) to assist in refining the language, improving clarity, and enhancing the structure of the text. The model was not involved in the generation of original scientific content, analysis, or interpretation of data. All outputs generated by the model were reviewed and edited by the authors to ensure accuracy and compliance with authorship responsibilities.

## Supplementary Information

Below is the link to the electronic supplementary material.

Supplementary Material 1

## Data Availability

The genome sequences analyzed in this study are publicly available from the NCBI database under BioProjects PRJNA806525, PRJNA526349, and PRJNA767482. The Escherichia coli K-12 reference genome (RefSeq NC\_000913.3) was used for comparative analyses. All data generated or analyzed during this study are included in this published article and its supplementary information files.
