# Supplementary material for: Structural modeling and docking analysis of canonical and novel resistance-associated missense mutations in Sudanese Escherichia coli
Source: Sci Rep. 2026 Feb 13;16:8995. doi: 10.1038/s41598-026-39491-7 (PMC12992559; doi:10.1038/s41598-026-39491-7)
Supplement: Supplementary file 1 — Supplementary Material 1 [file 41598_2026_39491_MOESM1_ESM.pdf]

Supplementary Tables S1 to S8

Structural Modeling and Docking Analysis of Canonical and Novel Resistance-Associated Missense Mutations in Sudanese Escherichia coli

Edison Eukun Sage, Sabah A. E. Ibrahim, Mohd Firdaus-Raih, Samah Omer A. Samhoon, Ahmed Abdelghyoum M. Mohamed, Tarig M. E. Ahmed, Moaaz M. Saadaldin, Omnia H. Suliman, Osama Mohamed, Sofia B. Mohamed*, Qurashi M. Ali

Table S1. PredictSNP analysis of missense mutations in Escherichia coli ParE

Protein	Mutation	PredictSNP (%)	PredictSNP result	MAPP (%)	PhD-SNP (%)	PolyPhen-1 (%)	PolyPhen-2 (%)	SIFT (%)	SNAP (%)	PANTHER (%)
ParE	R20H	87	Deleterious	76	88	74	65	79	72	NA
ParE	S458A	76	Deleterious	86	51	59	47	79	56	48
ParE	D250G	55	Neutral	51	73	67	87	79	62	48
ParE	I529L	63	Neutral	65	82	67	79	79	71	56
ParE	L416F	63	Neutral	57	61	67	61	66	58	65
ParE	T155N	74	Neutral	85	68	67	87	45	77	63
ParE	D596E	75	Neutral	41	83	67	87	84	83	56
ParE	V136I	83	Neutral	85	72	67	87	78	67	68
ParE	P231S	83	Neutral	71	68	67	87	81	61	71

PredictSNP values indicate expected prediction accuracy (%). The PredictSNP result corresponds to the consensus classification provided by the PredictSNP server. Individual tools (MAPP, PhD-SNP, PolyPhen-1, PolyPhen-2, SIFT, SNAP, and PANTHER) use tool-specific criteria. NA indicates unavailable predictions.

Table S2. PredictSNP analysis of missense mutations in Escherichia coli ParC

Protein	Mutation	PredictSNP (%)	PredictSNP result	MAPP (%)	PhD-SNP (%)	PolyPhen-1 (%)	PolyPhen-2 (%)	SIFT (%)	SNAP (%)	PANTHER (%)
ParC	L440R	87	Deleterious	57	77	74	55	79	72	65
ParC	S80I	64	Deleterious	41	68	59	74	46	56	67
ParC	E84G	55	Neutral	59	55	59	40	46	55	63
ParC	P577L	55	Neutral	68	73	59	40	53	67	NA
ParC	R229S	60	Neutral	41	61	67	71	53	61	70
ParC	L344R	74	Neutral	80	68	67	87	78	83	69
ParC	Q695L	74	Neutral	46	55	67	87	70	61	NA
ParC	K200N	75	Neutral	74	77	67	87	70	71	71
ParC	S57T	83	Neutral	70	78	67	87	78	83	71
ParC	D197E	83	Neutral	75	68	67	87	76	83	75
ParC	D309E	83	Neutral	80	83	67	79	87	83	71
ParC	D475E	83	Neutral	77	89	67	87	76	83	71
ParC	K716N	83	Neutral	NA	58	67	73	77	77	NA

PredictSNP values indicate expected prediction accuracy (%). PredictSNP result corresponds to the consensus classification provided by the PredictSNP server. Individual tools (MAPP, PhD-SNP, PolyPhen-1, PolyPhen-2, SIFT, SNAP, and PANTHER) use tool-specific criteria. NA indicates unavailable predictions

Table S3. PredictSNP analysis of missense mutations in Escherichia coli GyrA

Protein	Mutation	PredictSNP (%)	PredictSNP result	MAPP (%)	PhD-SNP (%)	PolyPhen-1 (%)	PolyPhen-2 (%)	SIFT (%)	SNAP (%)	PANTHER (%)
GyrA	G214V	87	Deleterious	86	88	74	81	79	56	48
GyrA	E477M	87	Deleterious	84	77	74	68	79	56	66
GyrA	N87Y	60	Neutral	57	59	67	87	79	50	71
GyrA	P872V	75	Neutral	46	78	NA	NA	67	71	NA
GyrA	L83S	83	Neutral	85	78	67	87	90	83	67
GyrA	N87D	83	Deleterious	85	83	67	87	90	77	65
GyrA	N87E	83	Neutral	75	68	67	87	65	67	67
GyrA	A669T	83	Neutral	70	51	67	76	87	77	71
GyrA	D678E	83	Neutral	85	72	67	87	87	83	67
GyrA	D741E	83	Neutral	77	68	67	87	90	83	NA
GyrA	A828S	83	Neutral	75	78	67	87	71	83	NA
GyrA	D867E	83	Neutral	77	72	NA	NA	76	83	NA
GyrA	V868S	83	Neutral	74	78	NA	NA	82	67	NA
GyrA	E871D	83	Neutral	65	89	NA	NA	87	71	NA
GyrA	P872S	83	Neutral	79	72	NA	NA	77	67	NA
GyrA	E873A	83	Neutral	71	83	NA	NA	76	58	NA
GyrA	E874D	83	Neutral	76	89	NA	NA	70	77	NA
GyrA	E875D	83	Neutral	NA	89	NA	NA	78	77	NA

PredictSNP values indicate expected prediction accuracy (%). PredictSNP result corresponds to the consensus classification provided by the PredictSNP server. Individual tools (MAPP, PhD-SNP, PolyPhen-1, PolyPhen-2, SIFT, SNAP, and PANTHER) use tool-specific criteria. NA indicates unavailable predictions.

Table S4. PredictSNP analysis of missense mutations in Escherichia coli GyrB

Protein	Mutation	PredictSNP (%)	PredictSNP result	MAPP (%)	PhD-SNP (%)	PolyPhen-1 (%)	PolyPhen-2 (%)	SIFT (%)	SNAP (%)	PANTHER (%)
GyrB	E185D	83	Neutral	79	78	67	87	90	83	67
GyrB	N492S	74	Neutral	57	58	67	87	90	67	67
GyrB	T618A	83	Neutral	NA	89	67	87	77	83	NA
GyrB	S635A	83	Neutral	NA	89	67	87	84	83	NA
GyrB	A655D	83	Neutral	NA	51	67	87	76	77	NA
GyrB	I663V	83	Neutral	NA	83	67	87	79	83	NA
GyrB	E703D	83	Neutral	NA	83	67	87	76	83	NA
GyrB	P715S	83	Neutral	NA	68	67	87	67	71	NA

PredictSNP values indicate expected prediction accuracy (%). PredictSNP result corresponds to the consensus classification provided by the PredictSNP server. Individual tools (MAPP, PhD-SNP, PolyPhen-1, PolyPhen-2, SIFT, SNAP, and PANTHER) use tool-specific criteria. NA indicates unavailable predictions.

Table S5. PredictSNP analysis of missense mutations in Escherichia coli RplV

Protein	Mutation	PredictSNP (%)	PredictSNP result	MAPP (%)	PhD-SNP (%)	PolyPhen-1 (%)	PolyPhen-2 (%)	SIFT (%)	SNAP (%)	PANTHER (%)
ribosomal protein L22 (rplV)	V71S	87	Deleterious	75	77	74	65	79	72	84
	I74N	87	Deleterious	75	73	74	65	79	72	76
	V76R	87	Deleterious	88	88	74	81	79	89	74
	D77R	87	Deleterious	62	86	74	56	79	81	67
	G79R	87	Deleterious	88	88	74	60	79	85	87
	K83A	87	Deleterious	84	86	74	68	79	72	77
	K83E	87	Deleterious	82	88	74	65	79	81	74
	M82H	72	Neutral	75	59	59	68	79	72	66
	S81E	61	Neutral	66	45	59	71	79	62	57
	E78R	55	Neutral	64	82	59	79	46	56	52
	T72Y	63	Neutral	41	55	67	74	79	50	69
	K73E	83	Neutral	71	72	67	87	76	71	57

PredictSNP values indicate expected prediction accuracy (%). PredictSNP result corresponds to the consensus classification provided by the PredictSNP server. Individual tools (MAPP, PhD-SNP, PolyPhen-1, PolyPhen-2, SIFT, SNAP, and PANTHER) use tool-specific criteria. NA indicates unavailable predictions.

Table S6. PredictSNP analysis of missense mutations in Escherichia coli RpoB

Protein	Mutation	PredictSNP (%)	PredictSNP result	MAPP (%)	PhD-SNP (%)	PolyPhen-1 (%)	PolyPhen-2 (%)	SIFT (%)	SNAP (%)	PANTHER (%)
RpoB	D516N	61	Deleterious	77	77	67	87	79	72	NA
RpoB	H526N	55	Deleterious	64	77	67	NA	79	81	56
RpoB	K439I	83	Neutral	79	58	67	87	68	55	57

PredictSNP values indicate expected prediction accuracy (%). PredictSNP result corresponds to the consensus classification provided by the PredictSNP server. Individual tools (MAPP, PhD-SNP, PolyPhen-1, PolyPhen-2, SIFT, SNAP, and PANTHER) use tool-specific criteria. NA indicates unavailable predictions.

Table S7. PredictSNP analysis of missense mutations in Escherichia coli RpoC

Protein	Mutation	PredictSNP (%)	PredictSNP result	MAPP (%)	PhD-SNP (%)	PolyPhen-1 (%)	PolyPhen-2 (%)	SIFT (%)	SNAP (%)	PANTHER (%)
RpoC	S590T	74	Neutral	71	58	67	87	43	61	71
RpoC	I908L	83	Neutral	85	83	67	87	90	63	70

PredictSNP values indicate expected prediction accuracy (%). PredictSNP result corresponds to the consensus classification provided by the PredictSNP server. Individual tools (MAPP, PhD-SNP, PolyPhen-1, PolyPhen-2, SIFT, SNAP, and PANTHER) use tool-specific criteria. NA indicates unavailable predictions.

Table S 8. MutPred2 analysis of the influence of missense mutations on molecular mechanisms

**Proteins**	**Mutations**	**Molecular Mechanisms (P-values <= 0.05)**	**Probability**	P-value
**RpoB**	D516N	Altered Disordered Interface	0.28	0.04
	H526N	Gain of Allosteric Site at R529	0.19	0.04
		Gain of Methylation at K527	0.14	0.02
**ParE**	D250G	Loss of Helix	0.28	0.03
		Gain of Strand	0.27	0.03
		Gain of Loop	0.27	0.02
		Altered Ordered Interface	0.25	0.02
		Loss of Allosteric Site at W251	0.21	0.04
		Altered Metal Binding	0.20	0.04
		Altered Transmembrane Protein	0.13	0.02
	S458A	Loss of Relative Solvent Accessibility	0.28	0.02
		Loss of Loop	0.27	0.04
		Gain of Allosteric Site at H462	0.24	0.02
		Gain of Catalytic Site at E460	0.11	0.03
**ParC**	L440R	Gain of B-factor	0.26	0.02
		Loss of Acetylation at K442	0.24	0.02
	S80I	Altered Ordered interface	0.28	0.03
		Gain of Relative solvent accessibility	0.26	0.03
		Gain of Catalytic site at H75	0.25	0.02
		Loss of Allosteric site at Y33	0.25	0.05
		Gain of Catalytic site at H75	0.20	0.03
		Loss of Allosteric site at Y33	0.18	0.05
	P577L	none	-	-
	E84G	Altered Metal binding	0.28	0.02
		Altered Ordered interface	0.28	0.05
		Loss of Relative solvent accessibility	0.24	0.05
		Loss of Allosteric site at Y33	0.23	0.02
		Loss of Catalytic site at D79	0.18	0.02
**GyrA**	G214V	Gain of Relative Solvent Accessibility	0.28	0.02
		Loss of Catalytic Site at T219	0.16	0.02
**ribosomal protein L22 (rplV)**	V71S	Altered Ordered interface	0.29	0.03
		Altered Ordered interface	0.28	0.04
		Loss of Relative solvent accessibility	0.27	0.02
		Gain of Acetylation at K73	0.20	0.04
	I74N	Altered Ordered interface	0.30	0.02
		Gain of Acetylation at K73	0.21	0.03
		Altered DNA binding	0.20	0.02
	V76R	Gain of intrinsic disorder	0.35	0.02
		Altered Ordered interface	0.29	0.03
		Loss of Relative solvent accessibility	0.29	0.01
		Gain of Acetylation at K73	0.22	0.03
		Altered Transmembrane protein	0.13	0.02
	D77R	Altered Ordered interface	0.30	0.02
		Loss of Relative solvent accessibility	0.26	0.03

		Altered DNA binding	0.23	0.01
		Gain of Acetylation at K73	0.21	0.03
	E78R	Altered Ordered interface	0.28	0.03
		Altered Disordered interface	0.28	0.01
		Gain of Acetylation at K83	0.23	0.02
		Loss of Allosteric site at K83	0.21	0.04
		Gain of Catalytic site at K83	0.12	0.03
	G79R	Loss of Relative solvent accessibility	0.29	0.01
		Altered Ordered interface	0.28	0.03
		Gain of Helix	0.28	0.03
		Loss of Allosteric site at R84	0.27	0.01
		Loss of Loop	0.26	0.05
		Altered Disordered interface	0.25	0.02
		Gain of Acetylation at K83	0.21	0.03
		Gain of Catalytic site at K83	0.12	0.03
	K83E	Altered Disordered interface	0.30	0.01
		Altered Ordered interface	0.29	0.03
		Loss of Relative solvent accessibility	0.26	0.03
		Altered Metal binding	0.22	0.03
		Gain of Catalytic site at R84	0.14	0.02
	K83A	Altered Ordered interface	0.30	0.02
		Altered Disordered interface	0.30	0.02
		Altered Metal binding	0.19	0.02
		Gain of Catalytic site at R84	0.18	0.02
		Loss of Relative solvent accessibility	0.27	0.02
	M82H	Altered Metal binding	0.35	0.01
		Altered Ordered interface	0.31	0.02
		Altered Disordered interface	0.28	0.04
		Gain of Relative solvent accessibility	0.25	0.04
		Gain of Allosteric site at R84	0.25	0.01
		Gain of Catalytic site at K83	0.12	0.03
	S81E	Loss of Relative solvent accessibility	0.27	0.02
		Gain of Helix	0.27	0.05
		Loss of Acetylation at K83	0.25	0.01
		Altered Disordered interface	0.20	0.04
		Loss of Methylation at K83	0.18	0.01
		Gain of Catalytic site at K83	0.12	0.03

MutPred2 predictions of molecular mechanisms affected by resistance-associated missense mutations. Only mechanisms with P-values ≤ 0.05 are reported. Probability scores indicate confidence in the predicted functional effect
